# Ectopic expression of the *flavonoid 3′,5′-hydroxylase* gene in *Ipomoea nil* induces the accumulation of delphinidin-based anthocyanins in the petals and inhibits their opening

**DOI:** 10.1186/s12870-026-09522-6

**Published:** 2026-07-22

**Authors:** Ton-Phuc Huynh, Chihiro Motoyama, Hiroshi Oshima, Naoko Kozai, Nagisha Yamate, Fumio Hashimoto, Keiichi Shimizu

**Affiliations:** 1 United Graduate School of Agricultural Sciences, Kagoshima University, Kagoshima, 890-0065 Japan; 2 Faculty of Agriculture, Kagoshima University, Kagoshima, 890-0065 Japan

**Keywords:** *Agrobacterium*, Anthocyanin, Biosynthesis, Campanula, Eustoma, Flower, Pigment, Petal opening inhibition, Transcriptome

## Abstract

**Background:**

Many commercially important ornamental species, such as rose, carnation, and chrysanthemum, lack blue flower varieties due to the absence of the *flavonoid 3′,5′-hydroxylase* (*F3′5′H*) gene, which is essential for delphinidin-based anthocyanin (Dps) biosynthesis pathway. Heterologous *F3′5′H* genes have been introduced into these species to enable Dps accumulation. However, incompatibility between heterologous F3′5′H and the host metabolic background has been reported, and certain gene–host combinations fail to efficiently produce Dps. This study aimed to evaluate whether *Ipomoea nil*, a model plant for anthocyanin biosynthesis studies, can serve as a platform for functional analysis of *F3′5′H*. Although *I. nil* lacks an endogenous *F3′5′H* gene, it naturally accumulates cyanidin-based blue pigments in its petals. Introducing Dps biosynthesis into this species provides an opportunity to investigate how Dps accumulation alters floral pigmentation. In this study, *I. nil* was engineered to produce Dps through ectopic expression of *F3′5′H* genes from four different plant species.

**Result:**

This study represents the first successful engineering of Dps biosynthesis in the genus *Ipomoea*. Transgenic *I. nil* lines expressing *F3′5′H* from *Eustoma grandiflorum*, *Campanula medium*, and *Viola* × *wittrockiana* showed high Dps contents, whereas the gene from *Gentiana triflora* resulted in low content. High Dps content was strongly correlated with a notable physiological phenotype characterized by inhibited petal cell expansion during flowering, thereby preventing the flowers from being fully open. RNA-Seq analysis revealed significant changes in the expression of genes involved in the salicylic acid signaling pathway, anthocyanin transport, oxidative stress response, and senescence-associated processes in petals with inhibited opening.

**Conclusion:**

This study suggests that *Ipomoea nil* cv. Violet can serve as a useful system for functional analysis of *F3′5′H*. In addition, high-level expression of the *F3′5′H* gene and the resulting high concentration of Dps were correlated with inhibited petal opening in this cultivar. RNA-seq analysis enabled the identification of genes whose expression changes were strongly correlated with the inhibition of petal opening. These findings provide important insights into the mechanisms underlying the inhibition of petal opening caused by altered pigment accumulation.

**Supplementary Information:**

The online version contains supplementary material available at https://doi.org/10.1186/s12870-026-09522-6.

## Background

**Fig. 1:**
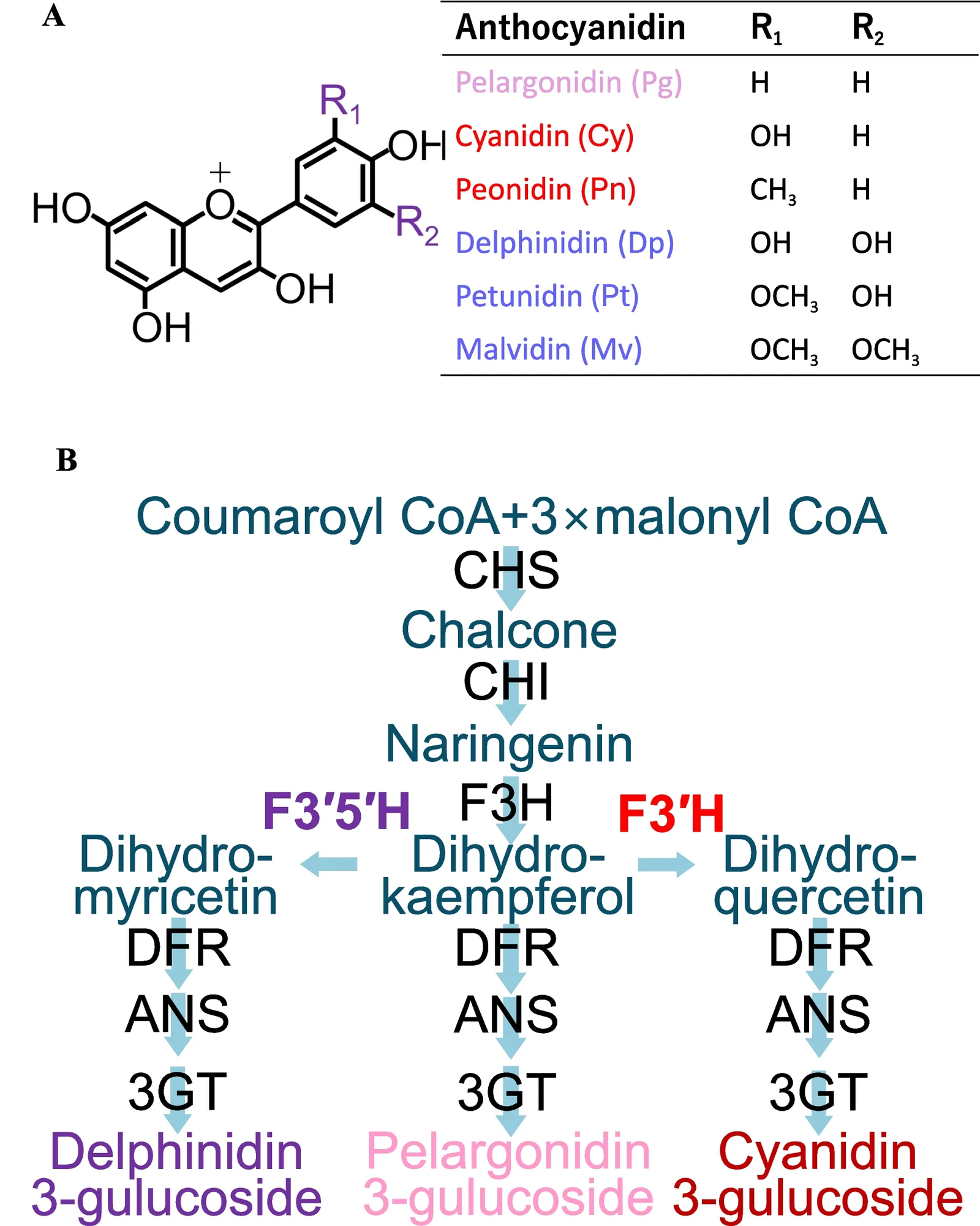
Anthocyanin synthesis pathway and anthocyanidin chemical structure. **A** General chemical structure and B-ring substitutions of major anthocyanidins. The core flavylium cation structure is shown (left) highlighting the variable substituent positions, while the accompanying table (right) details the functional groups distinguishing the six majors naturally occurring anthocyanidins. This anthocyanidin different with substitution of R_1_ and R_2:_ Pelargonidin (Pg) contains hydrogen atoms at both positions; Cyanidin (Cy) features one hydroxyl group and one hydrogen atom; Peonidin (Pn) is shown with a methyl group and a hydrogen atom; Delphinidin (Dp) possesses hydroxyl groups at both positions; Petunidin (Pt) contains one methoxy group and one hydroxyl group; and Malvidin (Mv) features methoxy groups at both positions. **B** Schematic representation of the general phenylpropanoid and anthocyanin biosynthesis pathways. The pathway illustrates the enzymatic steps, leading from phenylalanine to the main classes of anthocyanidins. Key enzymes include chalcone synthase (CHS), chalcone isomerase (CHI), and flavanone 3-hydroxylase (F3H). At the dihydrokaempferol (DHK) branch point, flavonoid 3′-hydroxylase (F3′H) and flavonoid 3′,5′-hydroxylase (F3′5′H) determine the B-ring hydroxylation pattern, that lead to the biosynthesis of cyanidin-based and delphinidin-based anthocyanins, respectively

The colors of several flowers are primarily determined by anthocyanins. These pigment colors are derived from their central chromophores, known as anthocyanidin. Anthocyanidins are classified into three main types based on their B-ring hydroxylation pattern: orange-red pelargonidin (Pg), red-magenta cyanidin (Cy), and blue-violet delphinidin (Dp). An alteration, such as methylation, converts Cy into peonidin (Pn) and Dp into petunidin (Pt) and malvidin (Mv) (Fig. 1A) [1]. Dp-based anthocyanins (Dps) (Fig. 1A), such as Dp, Pt, and Mv, are collectively responsible for most of the purple and blue floral pigmentations.

Anthocyanins are synthesized through the conserved phenylpropanoid and flavonoid pathways (Fig. 1B). The biosynthesis begins with the deamination of phenylalanine by phenylalanine ammonia-lyase, leading through several enzymatic steps to the formation of 4-coumaroyl-CoA. Chalcone synthase then catalyzes the first major step in flavonoid biosynthesis by condensing 4-coumaroyl-CoA with three molecules of malonyl-CoA to produce naringenin chalcone. Subsequent reactions involving chalcone isomerase (CHI) and flavanone 3-hydroxylase (F3H) yield dihydrokaempferol (DHK) [1, 2], at which point the B-ring hydroxylation pattern that primarily determines anthocyanin color is established. *Flavonoid 3′,5′-hydroxylase* (*F3′5′H*) gene encodes a cytochrome P450 monooxygenase that specifically hydroxylates the 3′ and 5′ positions of the flavonoid B-ring. This key step redirects metabolic flux toward the production of dihydromyricetin (DHM), the direct precursor for the synthesis of blue- and violet-colored Dp-based anthocyanins (Dps) [1]. Following hydroxylation, dihydroflavonol 4-reductase (DFR) catalyzes the reduction of dihydroflavonols such as DHM to colorless leucoanthocyanidins. These are then oxidized by anthocyanidin synthase (ANS) to form the corresponding colored anthocyanidins, which are subsequently glycosylated for stable vacuolar storage [1, 2].

Some economically important ornamental plants, such as roses, chrysanthemums, lilies, and carnations, cannot naturally produce blue flowers due to the absence of a functional *F3′5′H* gene that encodes the key enzyme required for the biosynthesis of Dps [1, 2]. Genetic engineering has become a primary strategy for developing novel, blue-flowered cultivars. The introduction of heterologous *F3′5′H* genes has led to the successful commercialization of Dp-accumulating transgenic carnations, phalaenopsis, and roses. However, this approach has some limitations because the degree of Dps accumulation, which varies significantly, depends on the functional compatibility between the introduced transgene and the metabolism of the host plant [3, 4]. Moreover, the precise molecular mechanisms underlying this compatibility are not fully understood, making the breeding of blue flowers a persistent challenge [5].

*Ipomea nil* (Japanese morning glory) is an annual climbing herb that has several advantages as a research material, owing to its short life cycle, efficient *Agrobacterium*-mediated transformation, and extensive genetic resources, including a fully sequenced genome [6–10]. When analyzing the function of F3′5′H using transgenic plants, both endogenous F3′5′H and flavonoid 3′-hydroxylase (F3′H), which competes with F3′5′H for the same substrate, can interfere with result interpretation. In contrast, *I. nil* lacks an endogenous *F3′5′H* gene, and mutant lines deficient in *F3′H* are readily available. Therefore, if the functionality of heterologous *F3′5′H* can be confirmed in *I. nil*, this species could serve as a powerful tool for functional analysis of this gene.

Although the host plant *I. nil* naturally shows blue coloration derived from cyanidin-based anthocyanins (Cys) and a high vacuolar pH, this coloration is often unstable and can be easily lost due to genetic mutations or physiological pH fluctuations [4]. Therefore, we hypothesized that engineering the accumulation of Dps would establish a pathway for a more robust, deep blue pigmentation. Furthermore, successful engineering of Dps biosynthesis in *I. nil* provides a critical foundational knowledge for future development of highly functional, health-promoting crops within the same genus, such as Dps accumulating sweet potatoes (*Ipomoea batatas*).

In this study, we aimed to evaluate whether transgenic *I. nil* can be used as a system for functional analysis of F3′5′H and to examine how Dps accumulation alters floral pigmentation in this species. Remarkably, in transgenic *I. nil* plants with high levels of *F3′5′H* expression and substantial Dps accumulation, a inhibition of petal opening was observed. To elucidate the molecular basis of this inhibition, comparative transcriptome analysis was performed on petals with inhibited opening.

## Methods

### Plant materials of Host *I. nil*

*I. nil* cv. Violet seeds were procured from Marutane Trading Co. Ltd. (Kyoto, Japan). The plant was cultivated in pots for seed production under a 16 h light/8 h dark cycle, 25 ± 3 °C temperature, and 27 μmol⋅m^−2^⋅s^−1^ PPFD fluorescent lighting. Suspension calli were initiated from immature embryos of the resulting plant according to the protocol previously described [11, 12].

### Construction of transgenic vector

**Fig. 2:**
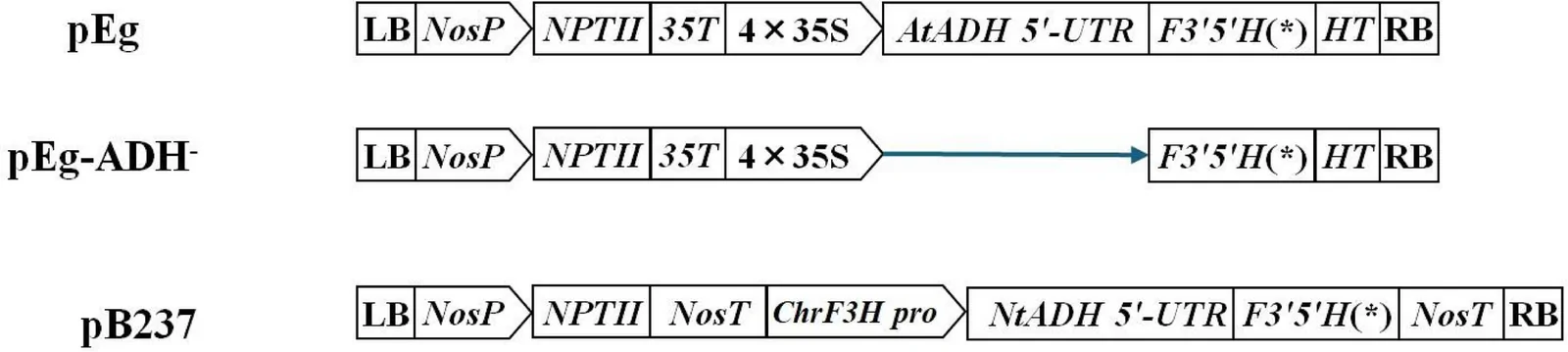
Vector structures containing the *Flavonoid 3',5'-hydroxylase *(*F3′5′H*) used for *I. nil* transformation. Each vector contains one *F3′5′H* gene from a specific species (*Eustoma grandiflorum*, *Campanula medium*, *Viola* *wittrockiana*, or *Gentiana triflora*). The T-DNA region of the binary vectors extend from the left border (LB) to the right border (RB) and includes the *nopaline synthase* gene *promoter* of Agrobacterium (*NosP*); a kanamycin resistance selectable marker (*NPTII, KmR*); *CaMV 35S* terminator (*35 T*); tetrameric enhancer array fused to the *CaMV 35S* promoter (*4* × *35S*), a *Arabidopsis thaliana Alcohol Dehydrogenase* 5′-UTR sequence (*AtADH 5′-UTR*), the *F3′5′H* coding region, and *Heat Shock Protein terminator* (*HT*). Two vector backbones were used: pEg (carrying *E. grandiflorum F3′5′H*), pEg ADH- (as *EgF3′5′H* but lacking the *A. thaliana ADH 5′-UTR*). Vector pB237 was built on back bone of pBI121 [4]. With structure similar to pEg however *MPTII* and *F3′5′H* were constructed with *nopaline synthase gene terminator* of Agrobacterium (*NosT*); *CAMV 35S* was replace *Chrysanthemum Morifolium F3H* Promoter (*ChrF3Hpro*); *AtADH5′-UTR* was replace with *Nicotiana tabacum Alcohol Dehydrogenase 5′-UTR sequence* (*NtADH 5′-UTR*). Elements outside the T-DNA include the *E. coli* origin of replication (Ori) and AmpR for bacterial selection. * The *F3′5′H* coding region was derived from one of the following species*: E. grandiflorum, C. medium, V.* × *wittrockiana,* or *G. triflora*

The sequence data for the construction of the binary vectors in this study (pKANre3AH, pEg, pEg-ADH^-^, pVw, pCm, and pGT) are provided as Supplementary Material 1 in formats that are compatible with SnapGene Viewer and ApE (A plasmid Editor). First, the Dps accumulation ability of three vectors containing the *E. grandiflorum F3′5'H* gene (*EgF3′5′H*) was compared. A schematic representation of the T-DNA region in the vector used in this study is shown in Fig. 2. To construct pEg, a binary vector, designated as pKANre3AH, was used. This vector is based on the pKAN43Ag8-GFP backbone [13] (Addgene #178,182), from which the GFP sequence was removed, and in which the translational enhancer and terminator were replaced with elements from pRI201AN (Takara Bio Inc., Otsu, Japan).

The *EgF3′5′H* gene was amplified via PCR from the cDNA isolated from the RV strain of *E. grandiflorum* [14] using KOD-Plus-Ver.2 polymerase (TOYOBO Co., Ltd., Osaka, Japan) and the primers listed in Supplementary Table S1. The amplified fragment was then inserted into the NdeI/SalI-digested pKANre3AH vector using the In-Fusion HD Cloning Kit (Takara Bio Inc.), resulting in the construction of pEg. The pEg-ADH^-^ construct was generated by inserting *EgF3′5′H* into XbaI/SalI-digested pKANre3AH using the same method as that for pEg construction, except that a different forward primer was used (see Table S1). The *EgF3′5′H* gene in pB237 [4] had already been incorporated into the vector.

To compare the Dps accumulation ability of *F3′5'H* genes from different plant species, the *F3′5'H* genes from *Campanula medium*, *Viola* × *wittrockiana*, and *Gentiana triflora* were used. The resulting constructs were named pCm, pVw, and pGt, respectively, and the *F3′5'H* genes in them were derived from plasmids pB244, pB247, and pB242 [4], respectively. The *F3′5'H* genes amplified from these vectors were integrated into pKANre3AH using the same method described above for pEg.

### Transformation

Transformation of *I. nil* was carried out following the protocol described by Takatori et al. [7] with minor modifications. The *Agrobacterium tumefaciens* strain AGL0, which harbored the vectors described above, was used to infect the calli of *I. nil* cv. Violet. The resulting transgenic calli were selected on a medium containing 120 mg/L of G-418 and one tablet/L of Augmentin250RS (GlaxoSmithKline K.K., Tokyo, Japan). After 3–4 weeks, the developing embryoids were transferred to EMG medium to promote shoot formation and root elongation. Regenerated plantlets were acclimatized in vermiculite for approximately one week before being moved to the soil. To confirm successful transformation, the regenerated plants were sprayed with a 300 mg/L kanamycin solution to test for resistance. Transgenic plants were grown for analysis and seed production under the same conditions as those for the wild type.

### High-Performance Liquid Chromatography (HPLC) analysis

Anthocyanidin contents were analyzed using HPLC, as described by Hashimoto et al. [15, 16] and Liu et al. [13]. Petals that had reached a developmental stage that corresponded to full bloom were subjected to freezing at − 80 °C, followed by pulverization in a 1% HCl/methanol solution for a duration of three days at 4 °C. Hydrolysis was performed using 2 N HCl at 100 °C for two hours. Extracts were filtered (0.45 μm) and analyzed using a Jasco HPLC system with a TSKgel ODS-80Ts QA Column. The mobile phase consisted of solvent A (1.5% phosphoric acid) and solvent B (1.5% phosphoric acid, 20% formic acid, 25% acetonitrile, and 5% tetrahydrofuran [THF]), with a gradient shift of over 35 min. Anthocyanin was detected at 525 nm using a UV–Vis detector to ensure precise quantification of the pigment concentration. In this study, the retention times varied among the HPLC runs; therefore, peaks were identified via co-injection with authentic anthocyanidin standards.

### RNA extraction, cDNA synthesis, RNA sequence, and Quantitative Real-Time RT-PCR (qPCR)

RNA was extracted from frozen plant tissues using the Total RNA Extraction Kit (RBC Bioscience) and then treated with DNase. PCR was used to confirm the complete digestion of the somatic DNA. Total RNA from each sample was standardized to a concentration of 50 ng/μL using a spectrophotometer. cDNA was synthesized using the PrimeScript™ II 1 st Strand cDNA Synthesis Kit (TaKaRa bio). Quantitative PCR (qPCR) was conducted using THUNDERBIRD SYBR qPCR Mix (TOYOBO) and an Applied Biosystems StepOnePlus™ Real-Time PCR System. A total of 10 μL reaction mixture was prepared for each reaction, containing 1 μL cDNA and 0.3 μL of specific primers (Supplementary Table 2). Relative expression levels were calculated using the ΔΔCt method. The transcript levels were normalized to the mitochondrial ATP synthase (*InATP*) gene as an internal reference [12, 17]. All the qRT-PCRs for each sample were performed in three biological replicates. Melting curve analysis of the amplicons was used to confirm the specificity of the PCR.

The RNA sequences were sent to the Seibutsugiken Bioengineering Laboratory for RNA sequencing to analyze changes in gene expression in *I. nil*. Total RNA quality was assessed using a Quantus Fluorometer (Promega) and a Fragment Analyzer System (Agilent Technologies). Libraries were prepared using the MGIEasy RNA Directional Library Prep Set (MGITech) and quantified using a Qubit 3.0 Fluorometer (Thermo Fisher Scientific). Sequencing was performed on a DNBSEQ-T7 platform with a paired-end of 150 bp read length. The raw reads were subjected to adapter trimming and quality filtering using Cutadapt and Sickle, followed by mapping to the *I. nil* reference genome using STAR. Expression levels were quantified using featureCounts and normalized using Transcripts Per Million (TPM). Differential gene expressions were analyzed using Python, and functional enrichment analysis was performed using GO annotations from the Asagao Database (Shigen Inc.) criteria for DEG with q.value < 0.05 and m.value > 1. To validate the RNA-seq results, nine DEGs were selected, and qRT-PCR was performed using primers designed from the sequences obtained from the database (http://viewer.shigen.info/asagao/) (Table S3), following the procedure described above.

### Observation of petal cells

The *I. nil* petals were harvested, trimmed, and embedded in 5% agarose. The cross-section was then cut using a thin blade and placed on a clean glass slide with water. A cover slip was placed over the section and examined under a light microscope at a magnification that allowed clear visualization of the cells.

### Statistical analysis

Statistical analyses were performed using the Tukey–Kramer multiple comparison test. Data are presented as mean ± standard deviation (SD). The numbers of biological replicates are indicated in the figure legends. Different letters above the bars indicate statistically significant differences at *p* < 0.05 based on Tukey–Kramer’s test. Relative expression of *F3′5′H* gene, as determined by RT-qPCR, was considered statistically significant at *p* < 0.05 based on ANOVA.

## Results

### Vector configuration is suitable for transgenic introduction of *F3′5′H* to *I. nil*

Transformation with pEg yielded 40 independent kanamycin-resistant plants, and HPLC analysis of petal anthocyanins revealed Dps accumulation in 23 of these plants (Table 1). As shown in Fig. S1A, the pEg construct yielded 22 transformants that accumulated up to six anthocyanidins (Pg, Cy, Dp, Pn, Pt, and Mv) and one transformant that accumulated three anthocyanidins (Figs. S1A and S1B). The highest Dps were 77.4% and 56.6%, respectively (Table 1). Notably, transformants with high Dps accumulation exhibited inhibited petal opening (Fig. S1A). Despite these abnormalities, no severe defects or morphological alterations were observed in other tissues across all transformants.

**Table 1:** Accumulation of Dps in petals of transgenic *Ipomea nil* plants expressing the *Eustoma Grandiflorum F3′5′H* gene with various vector configurations

Vector	Total Regenerated plants	No. of Dps-producing plants	Best (%)	Average (%)	SE
pEg	23	14	70.7	62.1 a	5.8
pEg-ADH^-^	7	7	67.3	51.8 a	14.6
pB237*	16	6	30.7	15.9 b	9.9

Percentage of Dps in total anthocyanins in petals. ^a, b^Different letters in the same column indicate significantly different means (*p* < 0.05), as determined by the Tukey’s test.

A total of six transformants were generated in the pEg-ADH^***-***^ lines, five of which accumulated Dps and six anthocyanidins (Fig. S1C). The maximum Dps reached 64%, with an average of 56.6% (Table 1). A similar petal inhibition was also observed (Fig. S1C).

The Dps contents in pEg and pEg-ADH^-^ transformants were significantly higher than those in the pB237 line, with 12 plants out of 18 transformants accumulating Dps with a maximum of 35% and an average of 14.5% (Table 1). Only three types of anthocyanidins (Pg, Pn, and Mv) were accumulated, with Pg being the major anthocyanidins, and no petal inhibition was observed (Fig. S1D). This result indicates that the combination of the *Cauliflower Mosaic Virus* (*CaMV* *35S)* and *HT* in vector configuration is effective for transgenic introduction of *F3′5′H* into *I. nil* for Dps accumulation; therefore, pEg is employed to construct a vector for subsequent experiments.

### Influence of *F3′5′H* from different plant species on Dps content in *I. nil*

Based on the above results, we constructed three additional types of vectors, pCm, pVw, and pGt, in which the *F3′5′H* gene of pEg was replaced with the corresponding *F3′5′H* derived from *C*. *medium*, *V.* × *wittrockiana*, or *G. trifloral*. These vectors were then introduced into *I. nil* through genetic transformation, and their effects were directly compared with those of pEg (Table 1). As shown in Table 2* [18], Dps content varied with the *F3′5′H* source; hence, individuals with accumulated Dps in each line were measured for comparison.

**Table 2:** Dps accumulation in petals of transgenic *Ipomea nil* expressing *F3′5′H* gene from different plant species

Vector	Total Regenerated plants	No. of Dps-producing plants	Best (%)	Average (%)	SE
pEg	23	14	70.7	62.1 b	5.8
pCm	17	8	94.2	85.9 a	7.9
pVw	21	10	83.8	52.2 b	29.2
pGt	12	7	10.8	7.1 c	3.2

Percentage of Dps in total anthocyanins in petals. ^a, b, c^Different letters in the same column indicate significantly different means (*p* < 0.05), as determined by the Tukey’s test

*This table was previously published in Huynh et al., 2023 [18]. The data are slightly different and updated for this study

The pCm line had the highest Dps content (86%, *p* < 0.05, n = 8), followed by *EgF3′5′H* (70.7%, *p* < 0.05, n = 11), and then *VwF3′5′H* (83.8%, *p* < 0.05, n = 10). These three lines had Dps as their primary pigment. In contrast, *GtF3′5′H* accumulated the lowest content (7%, *p* < 0.05, n = 7), with Pg as the primary pigment (Table 2) and no petal inhibition. These findings are similar to those of previous studies, which demonstrated species-dependent Dps variation in *F3′5′H* genes in transgenic chrysanthemum and rose [4, 5].

Transformants in the pCm line showed varied anthocyanidin compositions, ranging from five to six types of anthocyanidins (Figs. S1E and S1F); however, the pVw transformant composition was similar to that of the pEg composition, with most transformants accumulating the six types (Fig. S1G) and only one transformant accumulating three anthocyanidins (Fig. S1H). Notably, most pEg, pCm, and pVw transformants accumulated all the six anthocyanidins, whereas only three anthocyanidin types were detected in the pGt line (Fig. S1I).

The results for pVw have shown variability across the transgenic lines, which may be attributed to somatic chimerism, segregating gene silencing effects, or tissue culture stress. However, there are sufficiently large numbers of independent lines to confirm biological robustness. In this direction, further extensive research will be valuable to precisely measure *V. wittrockiana F3′5′H* function across a wider range of transgenic plants.

### *F3′5′H* gene expression and anthocyanidin content

To ensure stable inheritance and reliable evaluation of transgene and Dp accumulation, analyses were conducted on progeny T1. The presence of the transgene in the progeny of the transformants was confirmed via kanamycin spraying. For downstream analysis of the T1 generation, three independent lines were selected per construct (pEg, pVw, and pCm) that stably inherited the petal-opening-inhibition phenotype. To ensure true biological independence, each of these three lines was derived from a distinct T0 parental event. Furthermore, for each T1 plant evaluated, three separate petal samples were harvested and analyzed independently, serving as technical replicates. For pGt, analyses were conducted similarly using three independent lines, in which Dps accumulation was detected by HPLC. Transcript levels were calculated as the relative abundance normalized to *InATP*, with the internal control expression level set to 1.0.

**Fig. 3:**
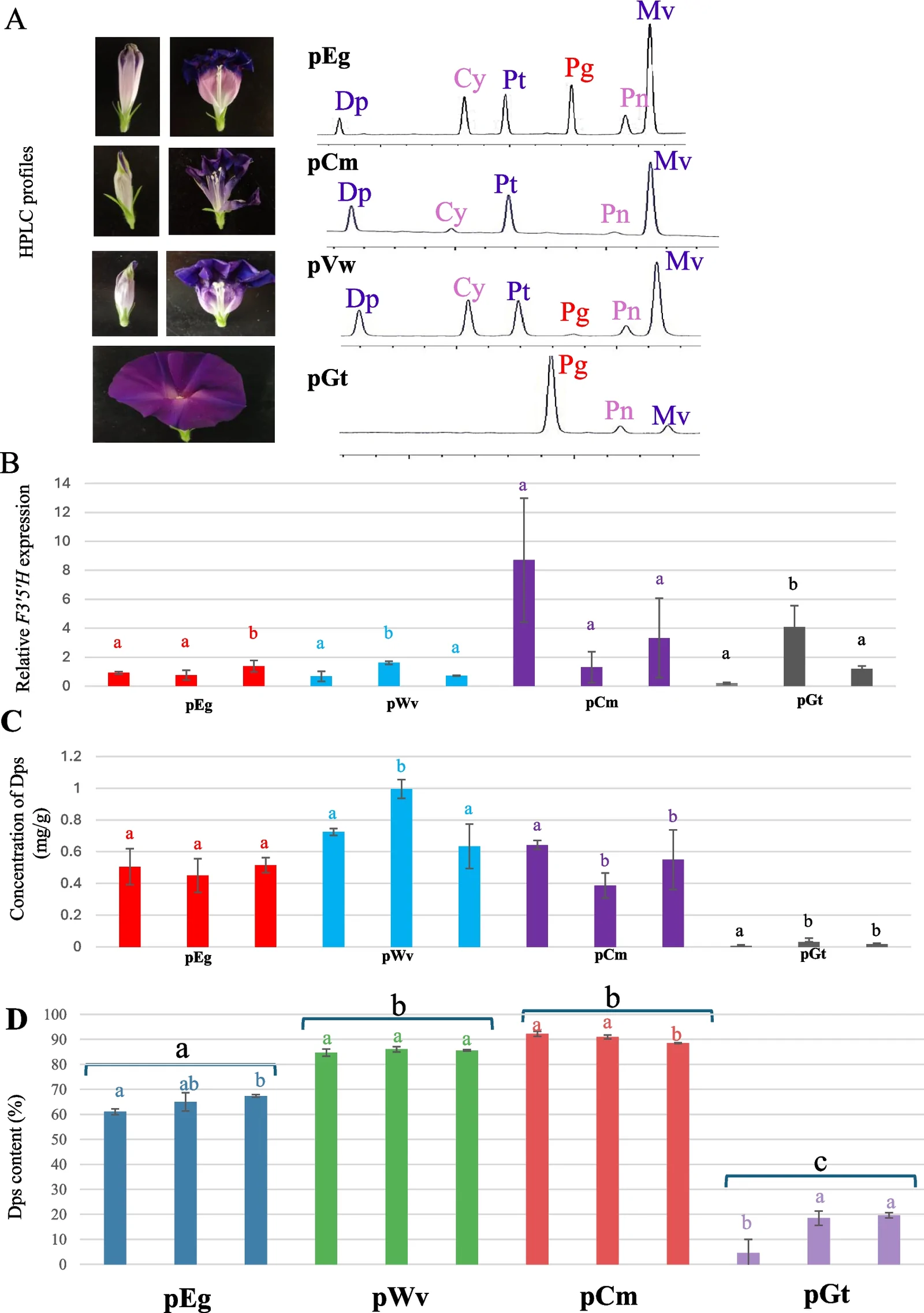
Characterization of transgenic *Ipomea nil* T1 progeny lines. **A** Flower phenotypes and High-Performance Liquid Chromatography (HPLC) profiles of anthocyanins in representative transgenic *I. nil* T1 progeny lines. Each line was transformed with an *F3′5′H* gene from one of four different species: *Eustoma grandiflorum*, *Campanula medium*, *Viola* *wittrockiana*, or *Gentiana triflora*. The chromatograms show peaks that correspond to various anthocyanidins: Dp (Delphinidin), Cy (Cyanidin), Pt (Petunidin), Pn (Peonidin), Mv (Malvidin), and Pg (Pelargonidin). Each peak was identified via co-injection with authentic anthocyanidin standards. **B** Relative expression levels of the introduced *Flavonoid 3',5'-hydroxylase *(*F3′5′H*) gene in three progeny individuals of each line. Expression levels are shown for lines pEg (*EgF3′5'H*), pWm (*VwF3′5'H*), pCm (*CmF3′5'H*), and pGt (*GtF3′5'H*). Expression levels were normalized to the internal control gene *InATP*, which was set to 1 for relative expression level analysis, and it is presented as relative transcript abundance. Data are presented as mean ± standard deviation (SD). Statistically significant difference between individuals within the same transgenic line was tested using Tukey HSD (*n* = 3, significant *p* < 0.05). **C** Accumulation of delphinidin-based anthocyanins (Dps) in the petals of the transgenic *I. nil* T1 progeny lines described in (B). The concentration of Dps is expressed as mg/g fresh weight. Data are presented as mean ± standard deviation (SD) for each individual in line. Statistically significant difference between individuals within the same transgenic line group was evaluated using Tukey’s HSD test (*p* < 0.05). Different letters indicate significantly different means (*p* < 0.05), as determined by the Tukey’s test. **D** Bar chart displays the Dps content (%) in progeny of lines pEg, pWv, pCm, and pGt. Data are presented as mean ± standard deviation (SD). Different lowercase letters above individual bars indicate significant differences between lines within the same construct, as determined using Tukey HSD test (*p* < 0.05). Letters above the brackets indicate significant differences between the overall means of the groups tested with Tukey HSD (*p* < 0.05)

The HPLC profiles of the T1 plants are shown in Fig. 3A. For T0 transformants, the Dps content in pCm was significantly different from that in pEg, pVw, and pGt (Table 2*). The progeny Dps content rate in Fig. 3 shows slightly different results: pCm (90.6%) was significantly higher than either pEg (64.5%) or pGt (14.2%) but not pVw (85.5%). Furthermore, pGt (14.2%) showed the lowest value, which was similar to that observed in the T0 generation Table 2. In each transgenic progeny line, no significant differences in appearance or HPLC profiles were observed among the individuals analyzed (Fig. S2). Significant variation in transgene expression was observed among individual plants within the pEg, pWv, and pGt lines (Fig. 3B). Significant differences in Dps contents were observed among the pEg, pCm, and pGt lines (Fig. 3D). Significant differences in Dps contents were also detected among individuals in the pWv, pCm, and pGt lines (Fig. 3C). In these lines, transgene expression and Dps accumulation showed a moderate degree of association, and a significant correlation was particularly detected in pVw (Fig. S4). However, Dps occupancy did not correlate with either of these variables.

### Inhibition of petal opening and transgene expression

**Fig. 4:**
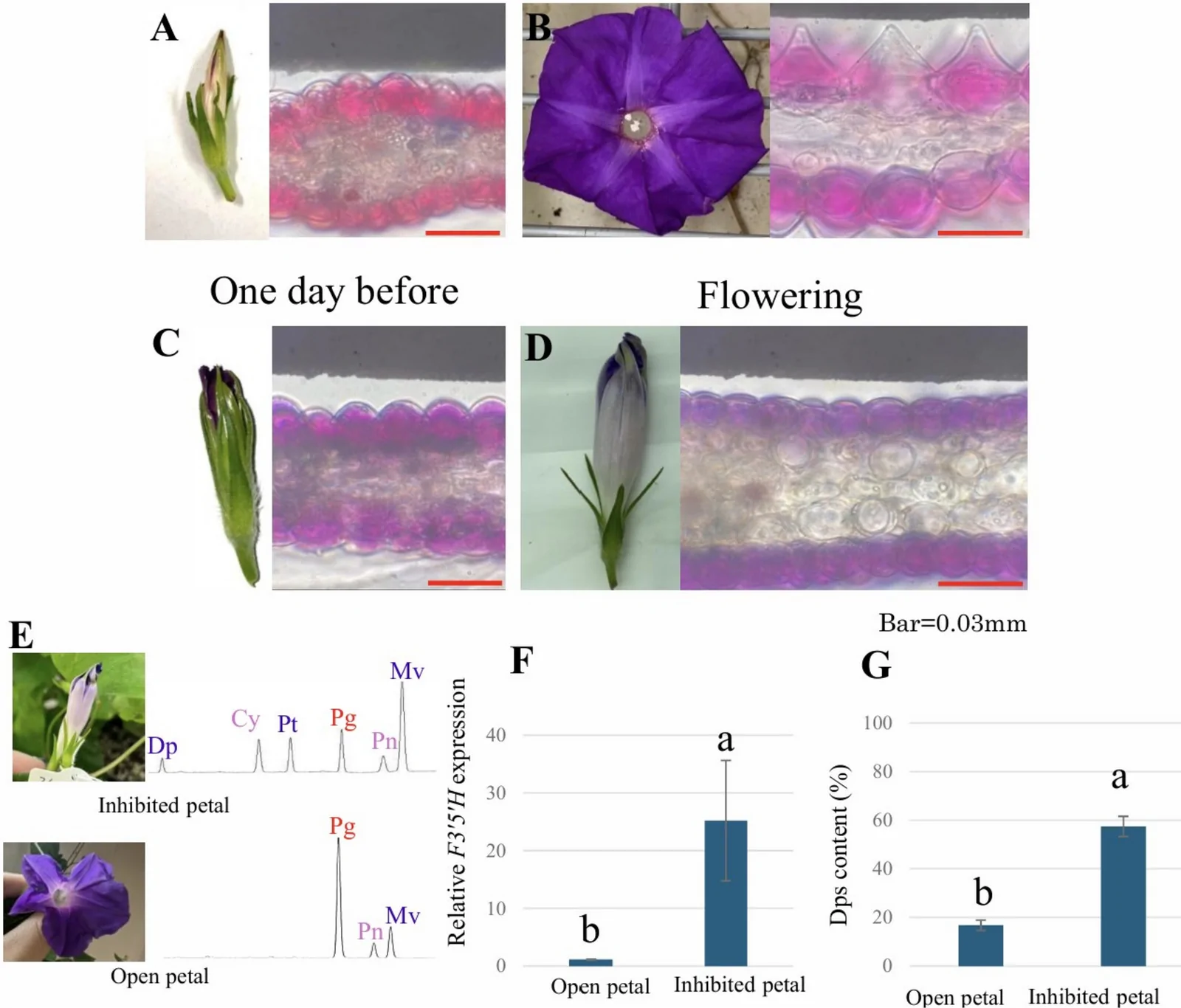
Microscopic analysis of petal cross-sections from a Delphinidin base anthocyanidin (Dps)-accumulating *Ipomoea nil* line across different developmental stages, including an inhibited flower opening phenotype. Red Bar = 0.03 mm. The figure displays: (**A**) One day before flower opening, with the corresponding petal cross-section showing initial pigment accumulation primarily in the epidermal cells. B* A fully open flower, with its petal cross-section revealing intense pinky-purple (Pg) pigmentation concentrated in the epidermal cells and clearly observed cells expansion. **C** A more developed but still closed bud, with its petal cross-section showing significant blue-purple pigmentation (Dps) in the epidermal cells. D* An inhibited bud, with its petal cross-section displaying blue-purple pigmented (Dps) epidermal cells. The cross-sections highlight the distribution and intensity of anthocyanin pigments within the petal cells, but no sign of cell expansion is observed. **E** Phenotype images and anthocyanidin profiles of opening and inhibited petals. **F** Relative expression level of *EgF3′5′H* in inhibited and open petals. The x-axis indicates relative expression level. Different lowercase letters above individual bars indicate significant differences between lines within the same construct, as determined using Turkey HSD test (*p* < 0.05). * This study is an extension of a report by Huynh et al. 2023 [17]. Fig. 3B and D in this paper were obtained from the report in Fig. 2

Microscopic cross-sectional analysis was conducted on the non-transgenic cv. Violet and Dps accumulated-inhibited pEg transgenic *I. nil* petals before and after bud opening (Figs. 4A–D). In non-transgenic host plants, conical epidermal cells exhibited normal physiological development characterized by significant expansion that increased cellular volume and led to the formation of a conical shape (Figs. 4A–B), indicative of petal opening [19, 20]. Conversely, the high Dps-accumulating line presented a peculiar phenotype characterized by non-expansion and non-opening of the petals. The epidermal cells in this line remained densely round with different pigment colors and failed to undergo the characteristic cellular expansion observed in the cv. Violet line (Figs. 4C–D). This developmental inhibition suggests that Dps accumulation correlates with petal opening inhibition in transgenic *I. nil*.

Most of the transgenic T0 generation plants for pEg, pVw, and pCm, with Dps content ranging from 55 to 85%, showed inhibited petal opening (Fig. S3A–C). Although some transgenic plants for pEg and pVm with lower Dps content (3–25%) produced fully open petals (Fig. S3D), these fully open petals were rarely observed on the same T0 generation individuals, that exhibited inhibited petals (samples VO43, VO38, EO18, and EO27 that were collected from the inhibited individuals V43, V38, E18, and E27, respectively). In contrast, all petals on the EO10 and VO25 transgenic plants were fully opened. As phenotypes in the T0 generation can potentially be affected by tissue culture effects or transgenic chimerism, we used the T1 generation for further analyses. In the T1 generation, we similarly observed the rare formation of fully open petals on same inhibited individuals of the pEg lines. Therefore, we used these samples to analyze the differences in gene expression and Dps content (Fig. 4E–G). As expected, the fully open petals showed relatively lower relative expression of the *F3′5'H* and significantly lower Dps content compared to inhibited petals. The major anthocyanidin in these samples was Pg, similar to that in the host *I. nil* cv. Violet, and Dp-type Mv were detected only at trace levels.

As the spontaneous formation of these fully open petals on the inhibited plants is exceptionally rare and unpredictable, sampling was inherently constrained. Specifically, two open and two inhibited petals were sampled simultaneously from the same two T1 individuals. To obtain a third sample pair, one fully open petal and one inhibited petal were collected from two distinct individuals. Owing to this rarity, it was not feasible to conduct technical replicates for this specific paired analysis. In contrast, no fully open petals were observed in the T1 generation of pVw and pCm.

### Transcriptome comparison of open and inhibited petals

**Fig. 5:**
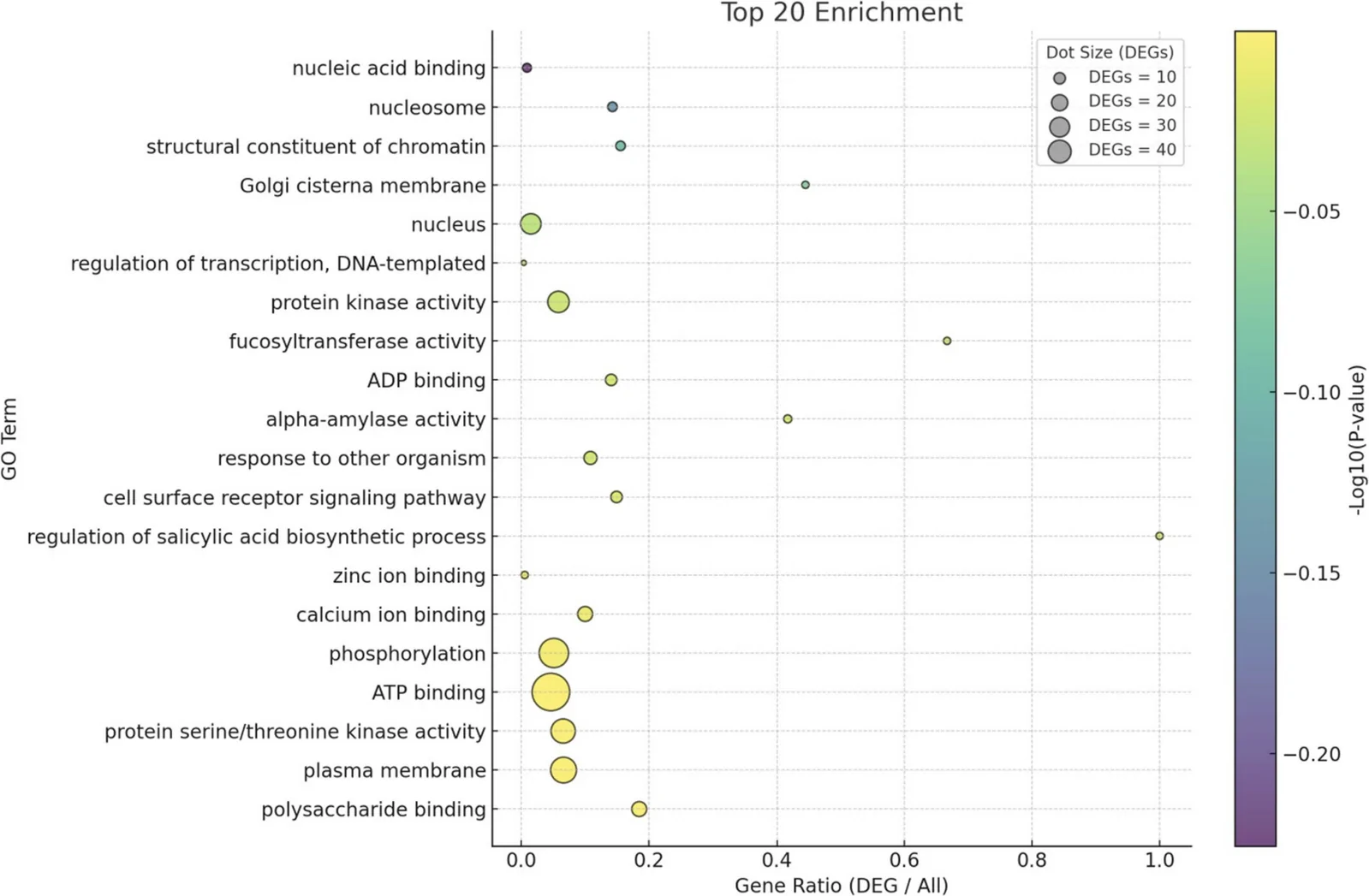
Gene Ontology (GO) term enrichment analysis of transgenic *Ipomoea nil* open and inhibited petals of T1 progeny. Gene Ontology (GO) enrichment analysis was performed on differentially expressed genes (DEGs) between open and inhibited petals of transgenic *I. nil*. The top 20 enriched GO terms are in three categories across Biological Process, Molecular Function, and Cellular Component. The x-axis is the Gene Ratio (number of DEGs annotated to a term divided by all DEGs), and terms are listed on the y-axis. Bubble size corresponds to the number of DEGs mapped to each term, and bubble color indicates enrichment significance *P*-value (darker = more significant). Abbreviation: DEG, differentially expressed gene

Both inhibited and open petals were observed in the progeny T1 of the pEg transformants, as described above. RNA sequencing of these petals revealed 913 differentially expressed genes (DEGs) between inhibited and open petals (Fig. 5, 6A). Gene Ontology (GO) enrichment analysis showed significant alterations, particularly the upregulation of terms related to salicylic acid (SA) biosynthesis (Fig. 5), suggesting a potential stress response [21, 22]. The highly upregulated genes were *SARD1-SAR DEFICIENT 1* (INIL01g04886 and INIL03g37226), *CBP60-Calmodulin-binding protein 60 A* (INIL03g20446), and *Calmodulin-binding protein 60 B* (INIL06g29912).

**Fig. 6:**
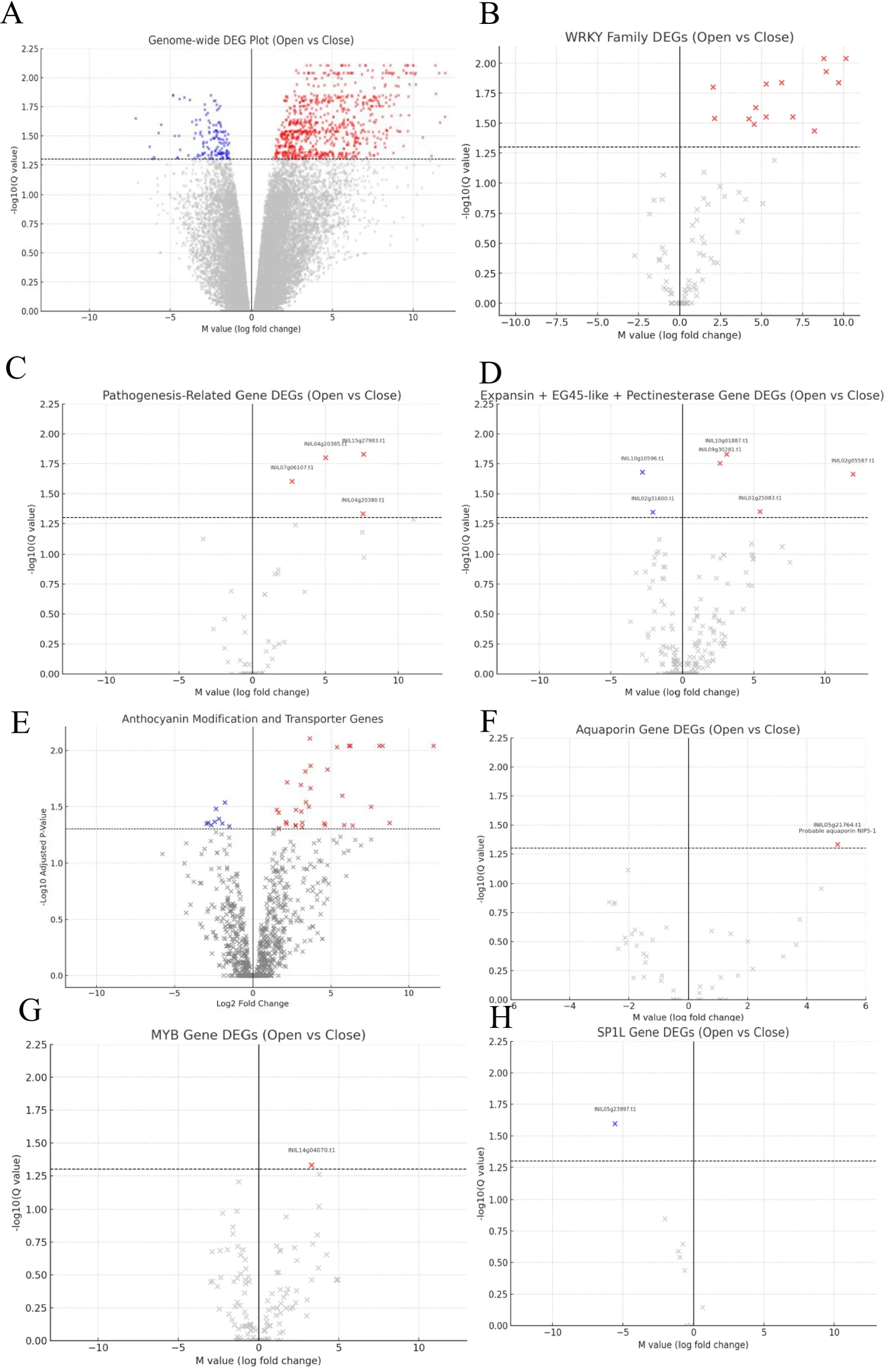
Transcriptomic analysis of differentially expressed genes between *Ipomea nil* open and inhibited petals. Differentially expression genes were analyzed based on log₂ fold change (x-axis) and statistical significance (adjusted *P*-value; y-axis). **A** Volcano plot showing the genome-wide distribution of differentially expressed genes (DEGs), **B** *WRKY* transcription factor family; **C** *Pathogenesis-related* (*PR*) genes; **D** Cell wall-modifying genes, including *expansin*, *EG45-like*, and *pectinesterase*; **E** Genes involved in anthocyanin transport and modification; **F** *Aquaporin* genes; **G** *MYB* transcription factors; H *SPL1* gene family. Red dots indicate significantly upregulated genes, blue dots represent significantly downregulated genes, and gray dots denote non-significant changes

Based on these findings, further analyses were conducted on the gene families associated with the SA pathway activation in other plant species, including the *Pathogenesis-Related Protein (PR)* family [23–25] and the *WRKY* co-transcription factor gene family (Van Verk MC et al., 2011 [26]). The results revealed four upregulated *PR* genes (Fig. 6C) and 14 upregulated *WRKY* genes (Fig. 6B), indicating a potentially strong activation of defense responses mediated by the SA pathway.

In addition, gene families associated with flower opening in *I. nil* were assessed. Aquaporins, which facilitate water transport required for cell expansion and petal movement [27, 28], were represented by 78 annotated genes, of which only one, *INIL05g21764* (*NIP5-1*), was differentially expressed (Fig. 6F). This gene was significantly upregulated in inhibited petals, with a log₂ fold change of above 5, suggesting its possible role in altered petal dynamics under inhibited opening conditions.

Thereafter, 96 *expansin* genes were analyzed along with the cell wall–modifying *pectinesterase* genes. Canonical *expansin* genes were not differentially expressed (Fig. 6D), but one upregulated gene encoding an EG45-like domain protein was observed. This protein may contribute to cell wall remodeling, which is critical for petal opening [29, 30]. Although several *pectinesterase* genes were also upregulated, *pectinesterase inhibitor* genes were downregulated by over four-fold, further supporting active cell wall loosening in the inhibited petals (Fig. 6D).

Dps were considered the primary metabolic compound affected in the inhibited petals; hence, the expression profiles of key anthocyanin-related genes, including *UGTs*, *GSTs/TT19-like*, *MATE/ABCC* transporters, *O-methyltransferases*, and *acyltransferases*, were analyzed. Nineteen genes related to anthocyanin transport and modification were differentially expressed (Fig. 6E; Supplementary Table S5), suggesting increased sequestration of anthocyanins into the vacuole. The potential toxicity of reactive oxygen species (ROS)-related genes was also evaluated. Five ROS-associated genes were upregulated (Table S5), suggesting the possible induction of somatic toxicity caused by oxidative-related species like anthocyanidin.

Finally, the gene families involved in structured petal development, such as *SPIRAL1-like (SP1L)* genes [31] and *Mixta*, a subfamily of *MYB* co-transcription factors associated with petal cell differentiation [32], were investigated. No *MYB* genes were significantly down-regulated or up-regulated in the inhibited petals (Fig. 6G). However, *INIL05g23997 (SP1L2)*, a member of the *SP1L* family, was differentially expressed (Fig. 6H). *SP1L* genes are essential for microtubule organization and directional cell growth [33], suggesting a possible role in affecting petal structure under inhibited conditions. Several genes related to petal inhibition were selected for validation using qRT-PCR, as shown in Fig. S6. The qRT-PCR results showed that the selected genes were upregulated.

### Top upregulated DEGs and comparison with petal development databases

To identify transcriptomic changes between open and inhibited petals in great detail, the top 20 upregulated DEGs were compared (Fig. 6A) using the interactive web-based database ipomoeanil.nibb.ac.jp/fpkm/, which visualizes the temporal transcriptomes of *I. nil* petals, created by Nakagawa et al. [28]. Among the top 20 upregulated DEGs in inhibited petals, most genes maintained very low levels throughout the development of petals, except for the unidentified INIL11g26672 gene (Table S3). Within these top 20 upregulated genes, several candidates were considered to be potentially associated with the inhibition of petal opening, including the INIL12g08473 (*MATE*) transporter and INIL06g37875 (*SAG39*) *senescence-associated gene 39*. Several genes presumed to be involved in petal inhibition were selected for validation using qRT-PCR, and the results are shown in Fig. S5.

## Discussion

### Vector design for Dps accumulation in transgenic *I. nil*

Careful selection of appropriate promoter is crucial for achieving robust transgene expression. The *CaMV 35S* promoter is widely used for constitutive gene expression in plant transformation [34]; however, its efficiency can be species-dependent. For example, a report showed that the *CaMV* 35S promoter is ineffective in some ornamental species such as chrysanthemum and gentian. In chrysanthemum, the native *F3H* promoter was found to be significantly more effective than the *CaMV* 35S promoter in influencing petal-specific expression [4].

In the present study, the vector constructs containing the *CaMV* 35S promoter (pEg and pEg-ADH^-^) were more effective in promoting Dp-based anthocyanin accumulation in *I. nil* than the pB237 vector, which contains the chrysanthemum *F3H* promoter. These constructs also differ in their terminator and other regulatory regions; therefore, enhanced expression cannot be attributed solely to the promoter. The result of this study, combined with several previous reports on successful flower color modification in *I. nil* using the *CaMV* 35S promoter [7, 9, 17, 35] suggests it as a highly effective promoter for modifying pigments in this species.

### Relative *F3′5′H* expression level and anthocyanin composition in transgenic plant

Noda et al. [4] reported that the high proportion of Dp in chrysanthemum ray florets was associated with the upregulation of the campanula *F3′5′H* transgene. This study revealed a significant positive correlation between transgene expression levels and Dps accumulations in petals of *I. nil* transformed with the *F3′5′H* gene from *V.* × *wittrockiana*. A similar, though not statistically significant, trend was observed for *I. nil*, which expresses *F3′5′H* genes derived from *E. grandiflorum* and *C. medium*. However, no correlation was detected between the transgene expression and Dps contents (%) in any of the transgenic lines. Katsumoto et al. [36] demonstrated that overexpressing *F3′5'H* was not enough to increase Dps content in rose metabolic engineering. This observation suggests that the proportion of Dps in transgenic *I. nil* is influenced not only by the expression level of the transgene but also by sequence-dependent functional properties of the *F3′5′H* variants.

### *F3′5′H* gene origins and complex metabolic flux in different host systems regulate anthocyanin composition

Engineering of novel flower colors via heterologous gene expression is highly dependent on the functional compatibility between the introduced exogenous gene and the host plant environment. The function of *F3′5′H* gene can vary with different species. For instance, *F3′5′H* genes from *V.* × *wittrockiana* and cineraria enabled moderate Dps accumulation in chrysanthemum, but the gene from campanula proved superior, yielding Dps levels that are as high as 95% [4]. Similarly, compared to other sources, the *C. medium*-derived *F3′5′H* was the most effective at generating Dps, when expressed in tobacco or rose [37, 38]). In contrast, the pGt progeny showed the lowest among all the lines (Fig. 3D) similar to pGt T0 (Table 2).

This variation suggests that functional compatibility is influenced by complex factors beyond simple gene expression, possibly including enzyme kinetics, substrate affinity, protein stability, and potential interactions within multi-enzyme complexes or metabolons. Furthermore, anthocyanidin composition is not solely affected by the activity of the introduced *F3′5′H*. The substrate specificity of downstream enzymes, particularly DFR, plays an important role. The DFR-B isozyme, primarily responsible for floral pigmentation in *I. nil*, is an asparagine (Asn)-type DFR, a class known for its broad substrate utility [39]. Previous studies have confirmed this critical role, as its knockout results in the complete loss of floral anthocyanins [8, 9]. This study demonstrates for the first time that the *I. nil* DFR-B efficiently reduces not only its native substrates DHK and dihydroquercetin (DHQ) but also DHM, the product of F3′5′H activity. This broad substrate acceptance explains why transgenic lines, such as those expressing the *E. grandiflorum F3′5′H*, were able to produce a full spectrum of anthocyanidins, including those derived from Pg, Cys and Dps.

### *I. nil* as a model for anthocyanin biosynthesis studies

Although established organisms such as *Arabidopsis thaliana* [40] and *Nicotiana* spp. [41] have been widely used in anthocyanin biosynthesis pathway, *I. nil* offers a unique and powerful advantages. Similar to other model systems, this system provides essential tools for genetic research, including a well-characterized genome, efficient transformation protocols, and a short lifecycle that favors experimental timelines. *I. nil* has a critical advantage over other models in the specific study of B-ring hydroxylation. The cv. Violet used in this study lacks a functional endogenous *F3'H*, and the species naturally lacks *F3′5'H*. This genetic background provides an ideal host for competition with native hydroxylases. In contrast, no available cultivated lines of *Nicotiana* spp. lack both *F3'H* and *F3′5'H* functions, making it difficult to assess the precise contribution of an introduced transgene without metabolic interference [42–44]. Although some *Petunia* × *hybrida* mutants lack hydroxylase functions, the cultivars are limited in terms of efficient genetic transformation. Moreover, the small and unpigmented flowers of *A. thaliana*, which is a widely adapted model for molecular genetics, make it unsuitable for research on visible pigment phenotypes.

### Petal opening and its inhibition in transgenic *I. nil*

This study revealed a significant and unexpected outcome: severe inhibition of petal opening in transgenic *I. nil* lines with high Dps content percentage (Fig. 4 and Fig. S3). Microscopic observations revealed that, unlike in wild-type plants, petal cells in these transgenic lines failed to expand, resulting in inhibited petal opening.

It is possible that the overexpression of transgenes driven by this consecutive *CaMV 35S* promoter causes abnormal somatic physiology, leading to petal inhibition. However, transgenic lines expressing the homologous *F3′5′H* gene from *G. triflora* (pGt, sharing 78.3% sequence identity with *E. grandiflorum F3′5′H*) under the identical *CaMV 35S* promoter exhibited normal petal opening alongside low Dps accumulation. This substantiates that developmental inhibition is triggered by specific metabolic products rather than the constitutive overexpression of homologous mRNA or protein.

Moreover, our preliminary experiments showed that overexpression of an *E. grandiflorum F3′5'H* variant with reduced enzymatic activity (due to a nucleotide substitution) did not lead to Dps accumulation and did not impair petal opening in *I. nil* (Huynh et al., in preparation). Therefore, our data indicate that the cause of petal inhibition is high Dps content rather than the constitutive expression of the transgene driven by the *CAMV 35S* promoter.

Aberrant accumulation of metabolic compounds leading to petal abnormalities has been reported in other species. For instance, the accumulation of Dp 3-glucoside causes petal abnormality in *P.* × *hybrida* [45], while mutations in *CHI* may result in chalcone accumulation and petal abnormality in *I. nil*. [17]. Similarly, petal malformation in *Torenia* has been linked to the accumulation of Cys or their metabolic precursors [46]. However, these phenotypes in *P.* × *hybrida*, *I. nil*, and *Torenia* were caused by recessive mutations, which makes it difficult to dissect the causal mechanisms by experimentally manipulating the expression of the genes responsible for pigment accumulation. In contrast, the transgenic *I. nil* system used in this study relied on targeted exogenous gene expression, offering a highly controllable model for elucidating the precise molecular mechanisms of pigment-induced petal abnormalities.

### Gene expression changes in inhibited petals

Salicylic acid (SA) is well known for its role in regulating plant defense mechanisms. In this study, GO enrichment analysis revealed that SA-associated processes were upregulated in petals with inhibited opening compared to petals with normal openings. Recent findings revealed the involvement of SA in cell wall remodeling during immune responses, as observed in *Pseudomonas*-infected plants, in which localized immune activation triggers structural changes in the cell wall [47]. Additionally, in phosphorus-deficient rice, SA mediates phosphorus remobilization by acting upstream of auxin and nitric oxide signaling [48].

The upregulation of genes associated with anthocyanin modification, transporters, and ROS may indicate cellular stress or efforts to sequester potentially harmful compounds in the vacuoles. Additionally, an anthocyanin-related MATE transporter gene was included among the top DEGs. In *A. thalia*, the *MATE* transporter *TRANSPARENT TESTA12* (*TT12*) functions as a proton-driven flavonoid importer into the vacuole [49, 50]. The expression of such genes in the inhibited petals suggests a protective response aimed at compartmentalizing newly synthesized or misprocessed flavonoids. Transgenic petals may attempt to conjugate and vacuole-sequester Dps anthocyanins, which are not naturally produced in *I. nil*. These findings suggest the presence of bottlenecks in Dps glycosylation, acylation, or transport processes. The accumulation of aglycones or aberrantly conjugated anthocyanin forms in the cytosol could lead to stress and then disrupt normal developmental processes.

This stress response likely activates key transcription factors, such as *SARD1* and *CBP60*, which in turn promote the biosynthesis of salicylic acid. Elevated SA levels can subsequently trigger the expression of *WRKY* and *PR* genes, which are known markers of SA-induced defense pathways. In this study, these genes were also significantly upregulated, indicating a potential association between stress-induced defense activation and inhibited petal opening phenotype. However, further studies are required to fully elucidate these triggering mechanisms.

**Fig. 7:**
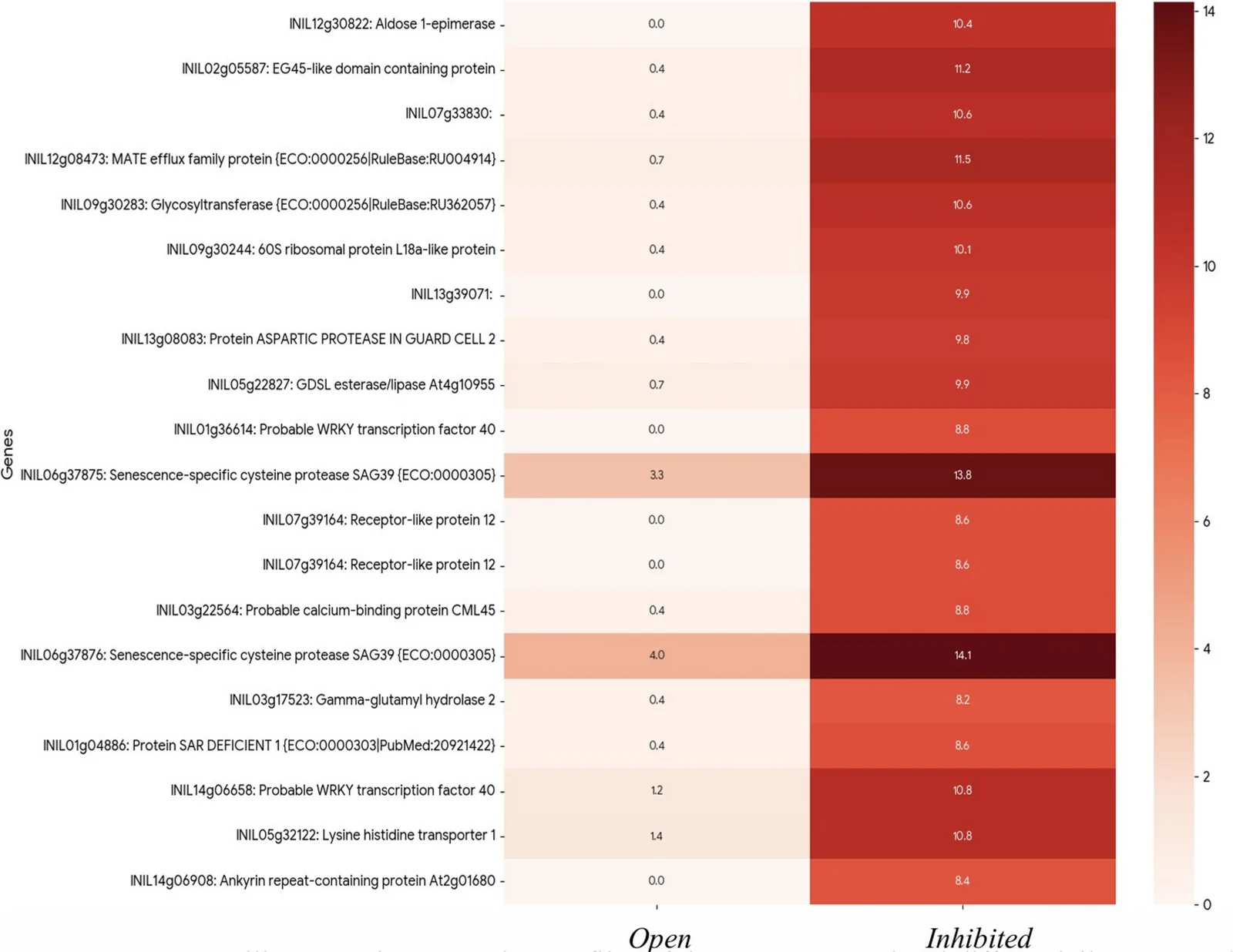
Heatmap and physiological characterization of gene expression in open and inhibited petals of *Ipomoea nil*. Heatmap illustrates the expression profiles of the top 20 upregulated differentially expressed genes (DEGs) identified in inhibited petals of *I. nil* compared to open petals. Each row represents one gene, labeled by transcript ID and putative annotation (where available). Columns represent biological replicates for mean value of open (*n* = 3) and inhibited (closed, *n* = 3) petal samples. Color intensity indicates raw expression levels (read counts), with the color scale ranging from low (white) to high (dark red). (E) Photographs of open and inhibited petals and their corresponding HPLC anthocyanin profiles. (F) Relative Flavonoid *3',5'-hydroxylase *(*F3′5′H*) gene expression levels of inhibited and open petals. Data for gene expressions are presented as mean ± standard deviation (SD). Different lowercase letters (a, b) indicate significantly different means (*p* < 0.05), as determined by Tukey’s test. (G) Delphinidin-based anthocyanin (Dp) content (%) in inhibited and open petals. Data for gene expressions are presented as mean ± standard deviation (SD). Different lowercase letters (a, b) indicate significantly different means (*p* < 0.05), as determined by the Tukey’s test

Among the top 20 upregulated DEGs (Fig. 7), two were classical stress responders. *HIPP26*, which responds to heavy metal (cadmium) exposure and drought stress [51], suggests the presence of abiotic or biotic stress signals in the inhibited petals.

Other significantly upregulated genes included those encoding *MATE* transporters and glycosyltransferases, further suggesting an attempt by plant cells to process and export cytosolic flavonoid pathway intermediates. Taken together, these correlative observations suggest that the accumulation of non-native compounds, such as Dps-related metabolites, may trigger a cellular defense response. However, direct causality and the precise molecular mechanisms should be elucidated through functional validation in future studies.

### Commercial and health significance of this study

Recent studies have demonstrated the high commercial and health value of anthocyanins. For example, the successful engineering of Dps accumulation in tomato fruits has enabled the production of highly functional, antioxidant-rich crops [52], and *I. nil* belongs to the same genus as sweet potato. Although sweet potato tuberous roots do not naturally accumulate Dps, the present study represents the first successful engineering of Dps biosynthesis in an *Ipomoea* species. Therefore, the genetic tools and functional insights from our model system provide a valuable foundation for engineering Dps-accumulating sweet potatoes with significant nutritional, health, and commercial value.

## Conclusion

This study reveals that the expression of multiple *F3′5′H* genes from different species in *I. nil* enables the production of Dps not naturally present in this species. These findings demonstrate that *I. nil* is a promising model system for investigating the functional role of F3′5′H in anthocyanin biosynthesis. The species origin of F3′5'H significantly influenced the anthocyanin composition in transgenic plants, indicating that its evolutionary divergence of *F3′5′H* contributes to distinct flavonoid metabolic outcomes. Notably, transgenic plants with high Dps exhibited restricted petal cell expansion during flowering. RNA sequencing results revealed the activation of detoxification- and defense-related pathways, suggesting that excessive accumulation of Dps pathway flavonoids may trigger stress responses that interfere with normal petal development. Although the specific genes directly responsible for petal abnormality were not identified in this study, a group of gene expression fluctuations that strongly correlated with this phenotype was identified. These findings provide a crucial foundation for elucidating the molecular mechanisms underlying petal abnormalities and underscore the broader significance of this research.

## Supplementary Information

Supplementary Material 1.

Supplementary Material 2.

## Data Availability

The datasets used and/or analyzed during the current study are available from the corresponding author on reasonable request.

## Abbreviations

35S: Cauliflower mosaic virus 35S promoter
ANS: Anthocyanidin synthase
Asn: Asparagine
*AtADH*: 5′-Untranslated region of the *Arabidopsis thaliana* alcohol dehydrogenase gene
cDNA: Complementary DNA
CHI: Chalcone isomerase
Cm: Campanula medium
Cy: Cyanidin
Cys: Cyanidin-based anthocyanins
DEGs: Differentially expressed genes
DFR: Dihydroflavonol 4-reductase
DHK: Dihydrokaempferol
DHM: Dihydromyricetin
DHQ: Dihydroquercetin
Dp: Delphinidin
Dps: Delphinidin-based anthocyanins
EC: Inhibited petal cells (Experimental Condition/Inhibited)
Eg: *Eustoma grandiflorum*
EO: Opening petal cells (Experimental Condition/Opening)
F3H: Flavanone 3-hydroxylase
F3′H: Flavonoid 3′-hydroxylase
F3′5′H: Flavonoid 3′,5′-hydroxylase
Gt: Gentiana triflora
HCl: Hydrochloric acid
HPLC: High-Performance Liquid Chromatography
HT: Arabidopsis heat shock protein 18.2 gene terminator
Mv: Malvidin
NOS: Nopaline synthase gene terminator
NtADH: 5′-untranslated region of the Nicotiana tabacum alcohol dehydrogenase gene
Pg: Pelargonidin
Pn: Peonidin
PPFD: Photosynthetic Photon Flux Density
PR: Pathogenesis-related
Pt: Petunidin
qPCR: Quantitative real-time reverse transcription-polymerase chain reaction
RNA: Ribonucleic acid
ROS: Reactive oxygen species
SA: Salicylic acid
SP1L: SPIRAL1-like
THF: Tetrahydrofuran
TPM: Transcripts Per Million
UV: Ultraviolet
Vw: Viola × wittrockiana

## Declarations

This manuscript is an extended and revised version of our conference paper published in Acta Horticulturae (Huynh et al., 2023) [18]. This submission includes additional experiments, expanded datasets, and new analyses that were not part of the original publication.
