# Supplementary material for: Ectopic expression of the flavonoid 3′,5′-hydroxylase gene in Ipomoea nil induces the accumulation of delphinidin-based anthocyanins in the petals and inhibits their opening
Source: BMC Plant Biol. 2026 Jul 22;26:1784. doi: 10.1186/s12870-026-09522-6 (PMC13673924; doi:10.1186/s12870-026-09522-6)
Supplement: Supplementary file 1 [file 12870_2026_9522_MOESM2_ESM.pdf]

Supplemental information

Supplemental material 2: Additional file 2 Figure and Table

Fig. S1

A

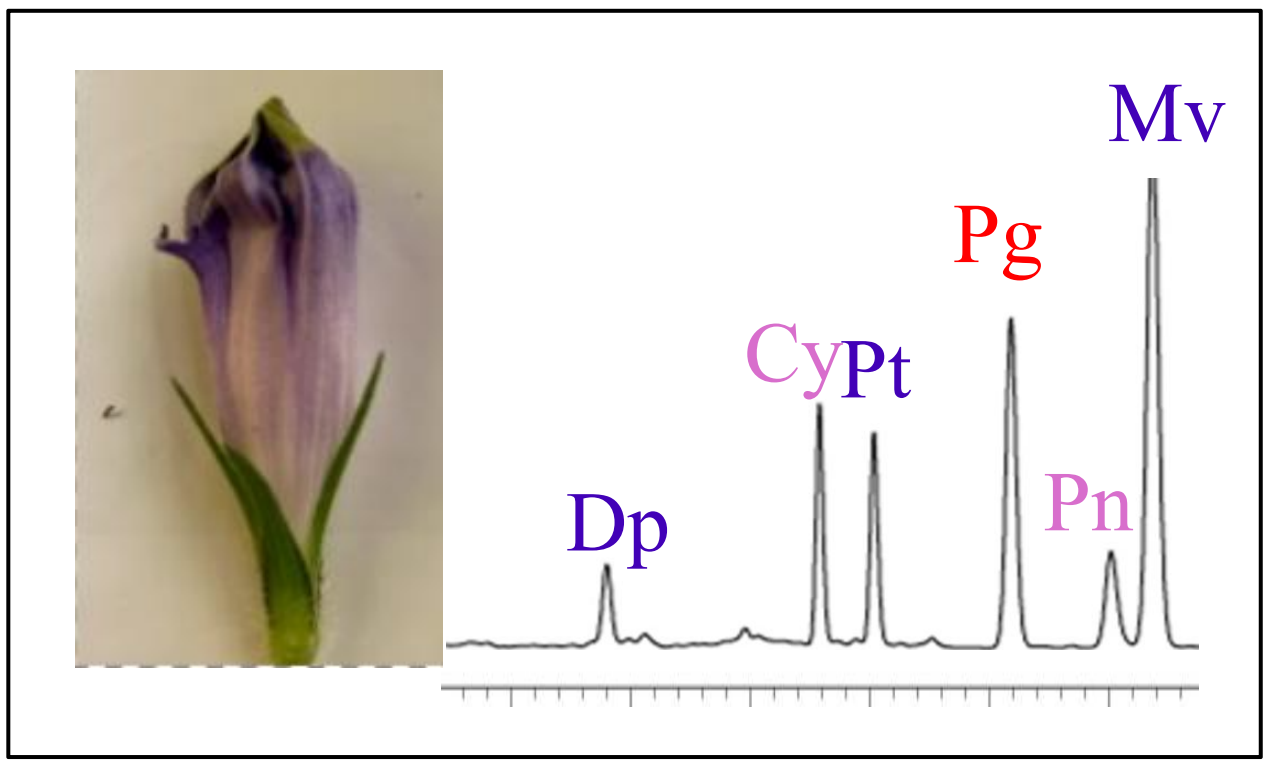

pEg-transformants with 6 anthocyanidins composition

B

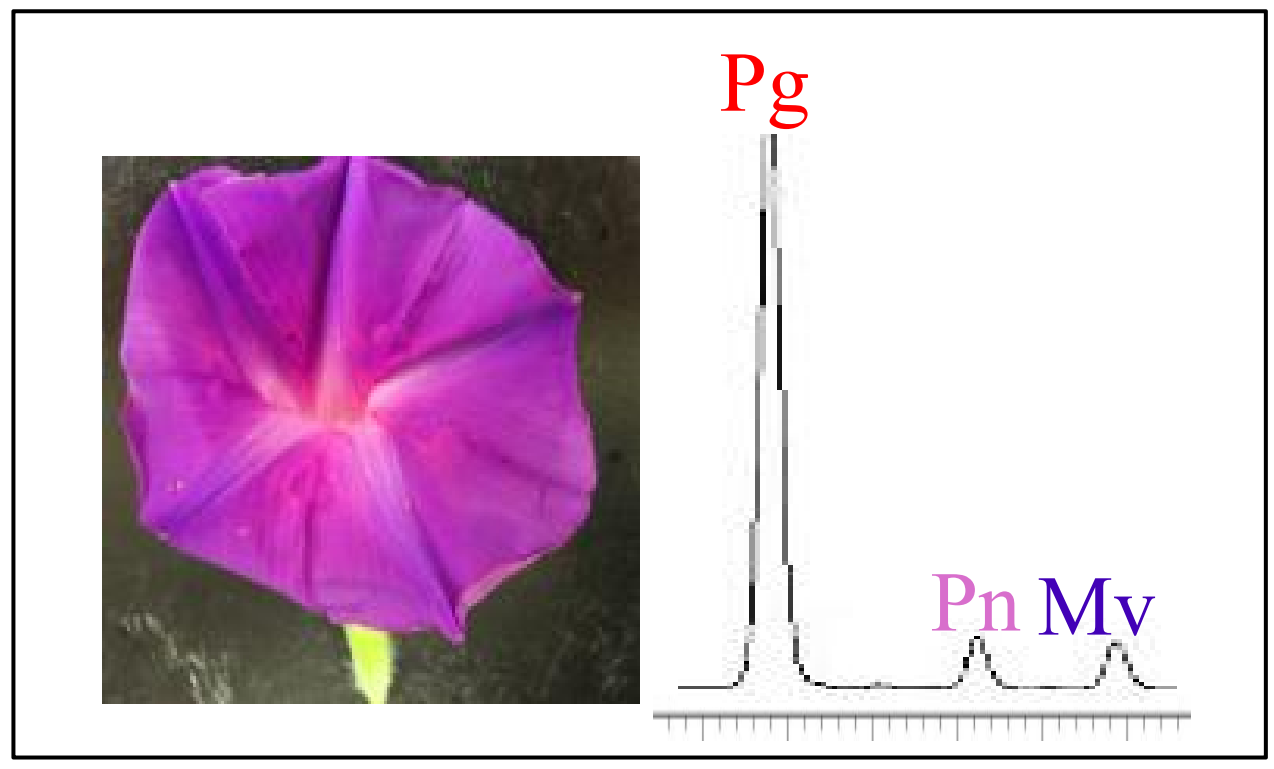

pGt transformants with 3 anthocyanidins composition

C

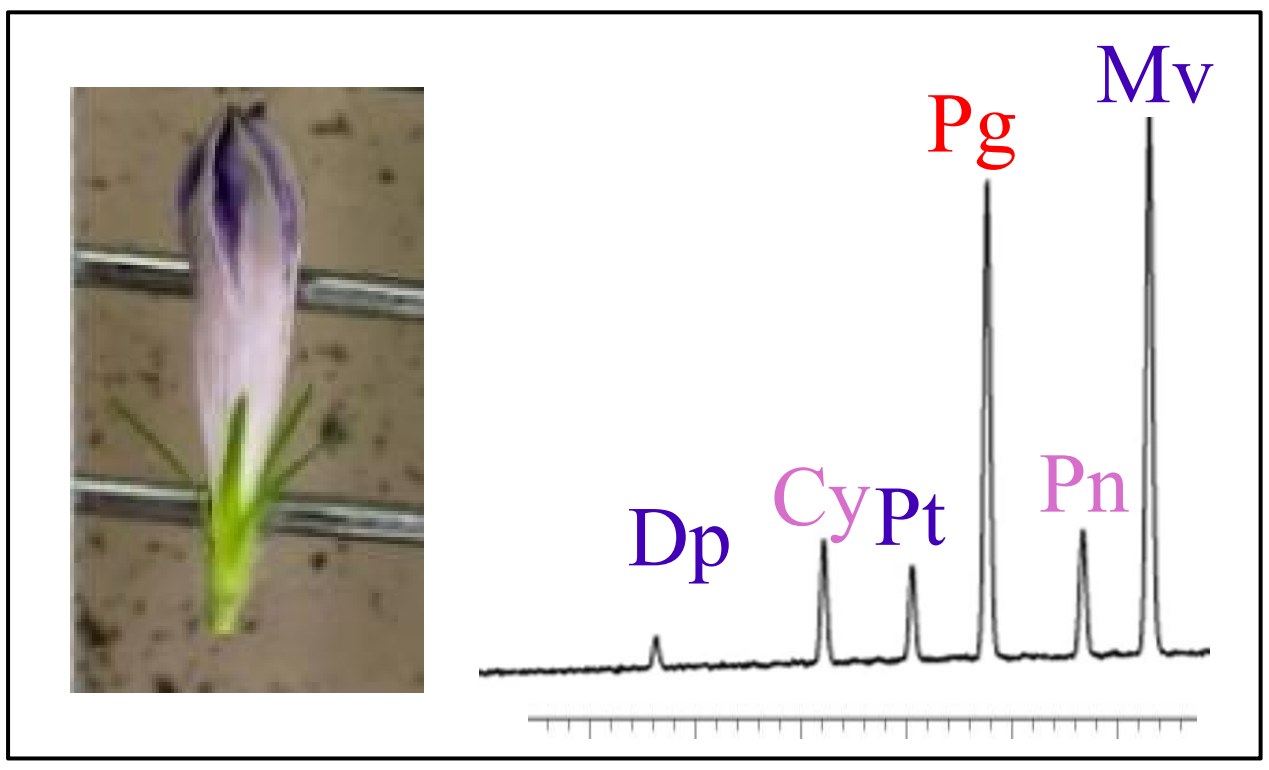

pEg-ADH-transformants with 6 anthocyanidins composition

D

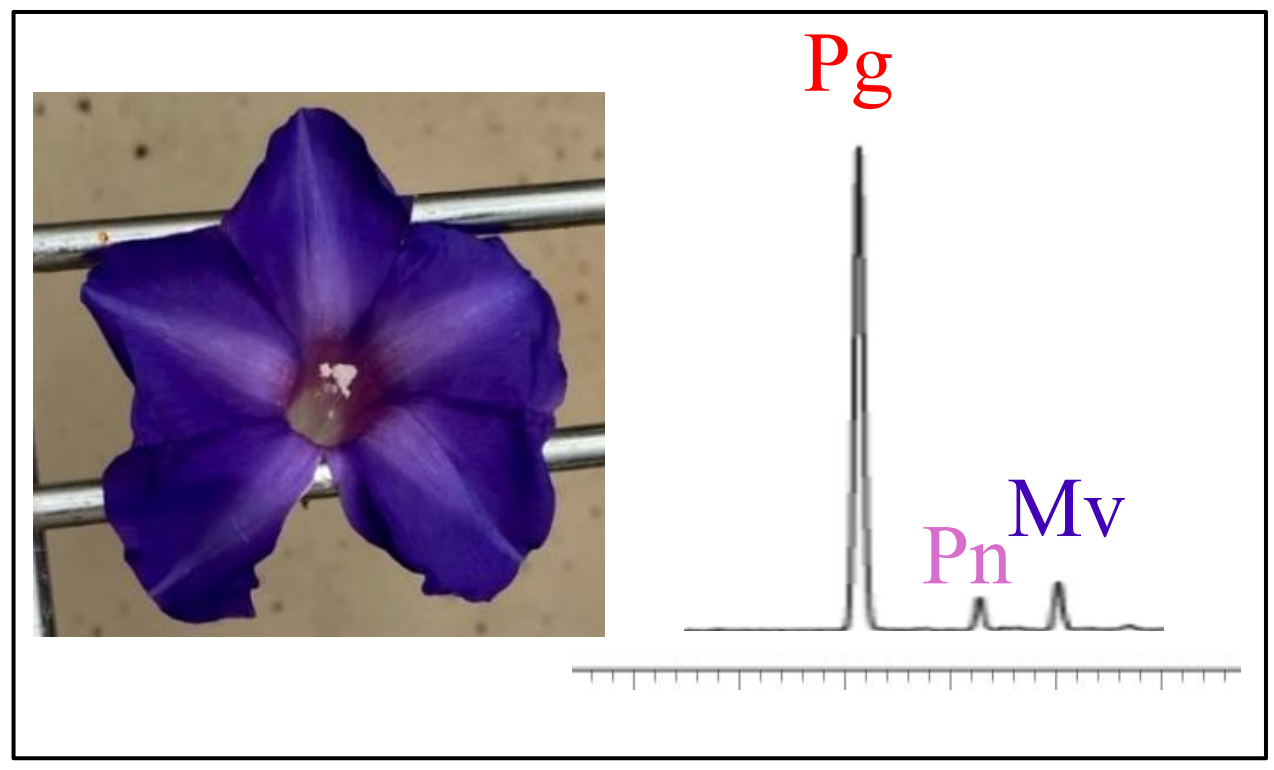

pB237 transformants with 3 anthocyanidins composition

E

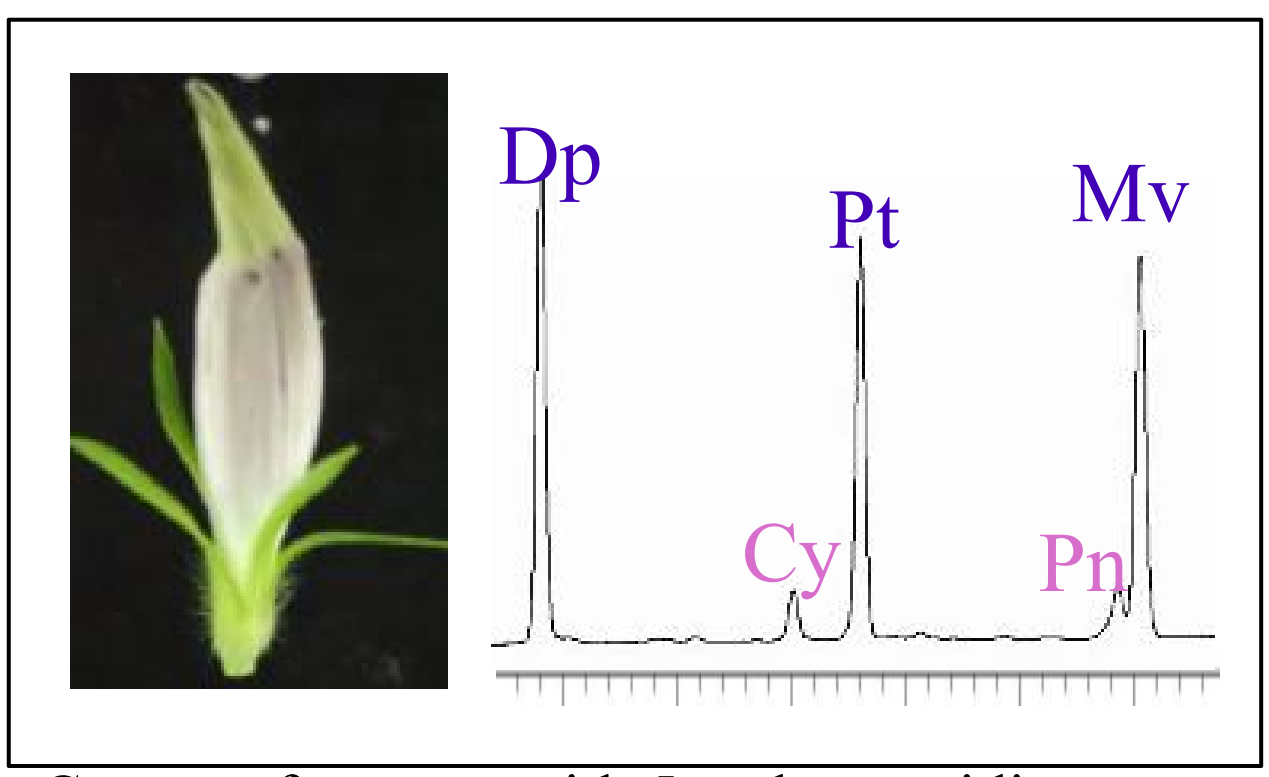

pCm transformants with 5 anthocyanidins composition

F

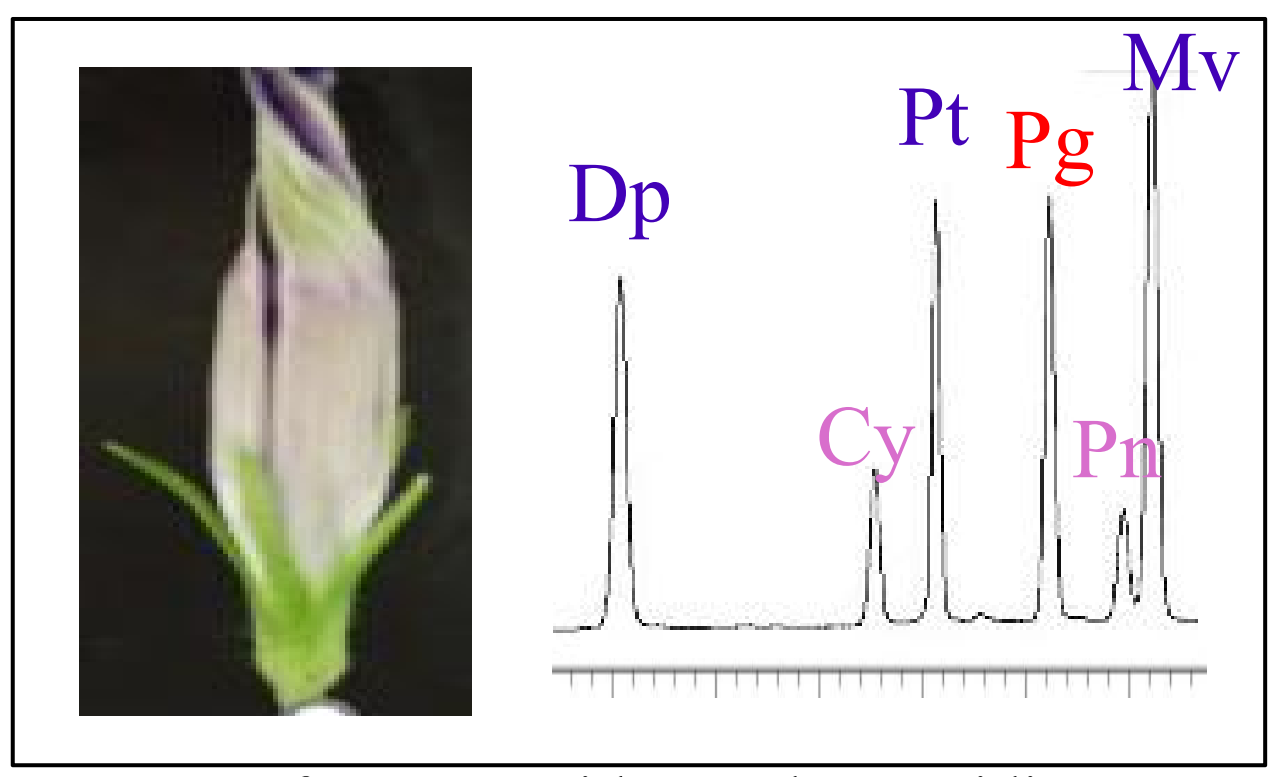

pCm transformants with 6 anthocyanidins composition

G

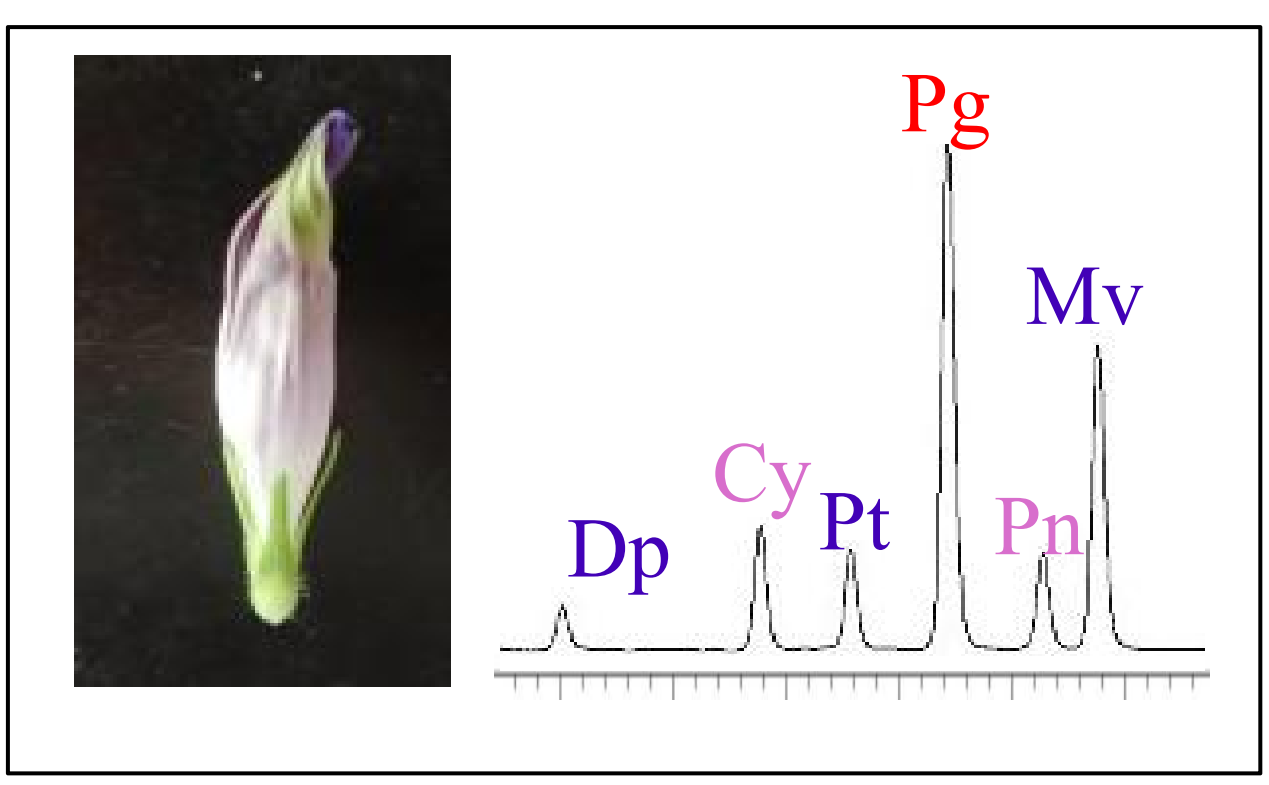

pVw transformants with 6 anthocyanidins composition

H

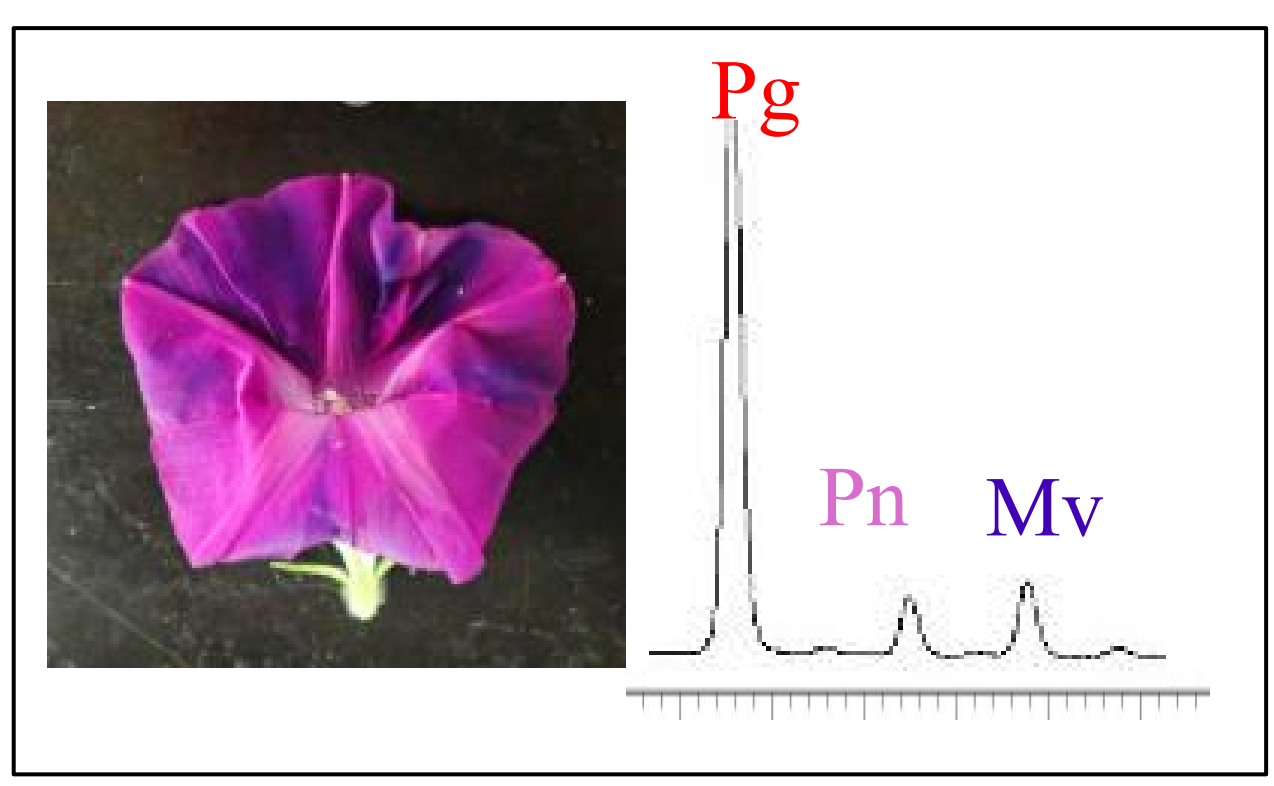

pVw transformants with 3 anthocyanidins composition

Fig. S1 Phenotype picture and HPLC chromatograms showing anthocyanidin profiles in the petals of T0 transformant. The detected anthocyanidins are labeled as follows: Dp, delphinidin; Cy, cyanidin; Pn, peonidin; Pg, pelargonidin; Pt, petunidin; Mv, malvidin. T0 of pEg has 1 varied HPLC compositions: (A) that accumulated 6 type anthocyanidins. T0 of pGt have 1 HPLC compositions: (B) that accumulated 3 types of anthocyanidins. T0 of pCm have 2 varied HPLC compositions: (E) that accumulated 5 type anthocyanidins and (F) that accumulated 3 types of anthocyanidins. T0 of pWm have 2 varied HPLC compositions: (G) that accumulated 6 type anthocyanidins and (H) that accumulated 3 types of anthocyanidins. Each peak was identified by co-injection with authentic anthocyanidin standards.

Fig. S2

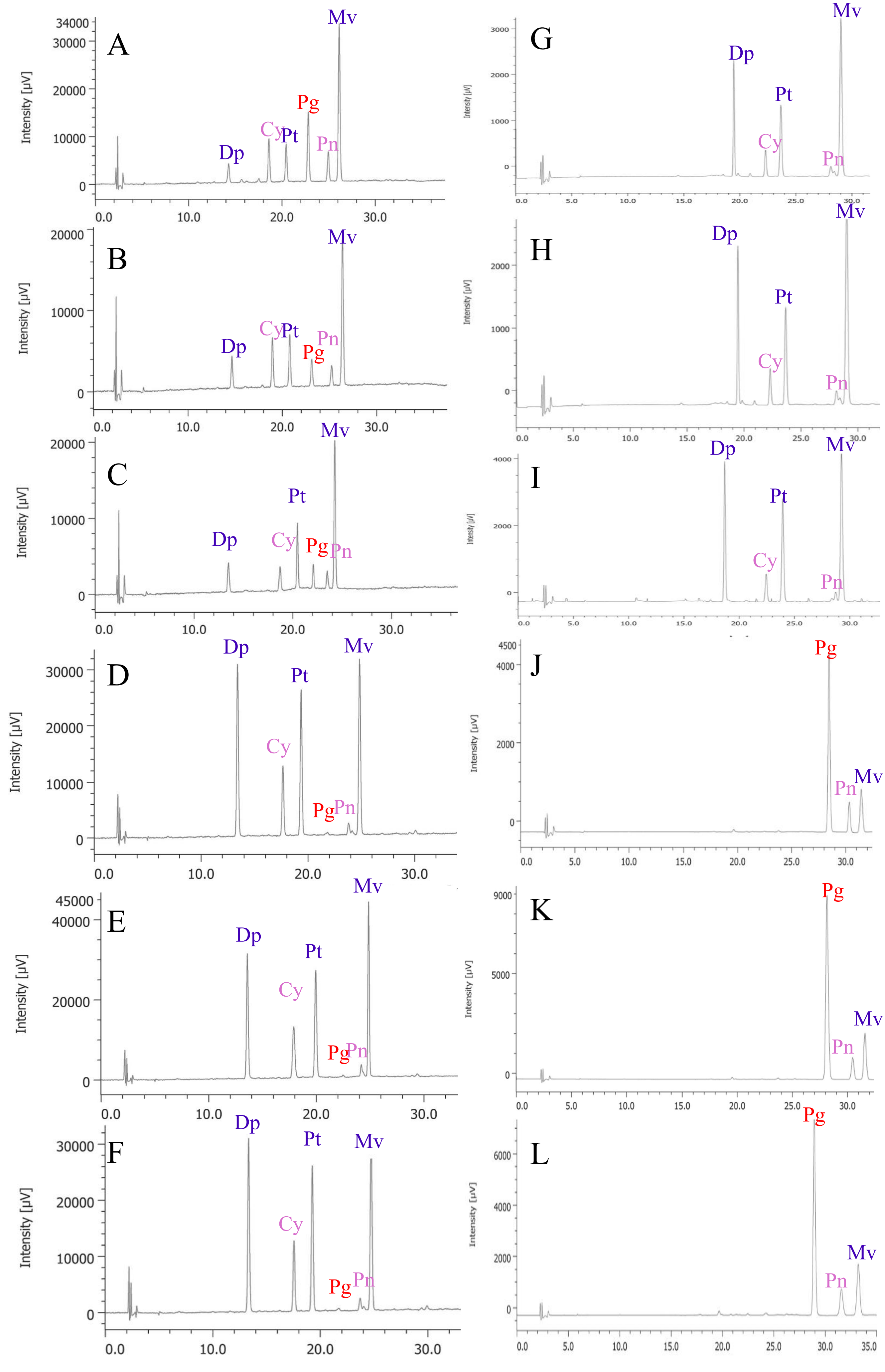

Fig. S2 HPLC chromatograms showing anthocyanidin profiles in the petals of T1 progeny. The detected anthocyanidins are labeled as follows: Dp, delphinidin; Cy, cyanidin; Pn, peonidin; Pg, pelargonidin; Pt, petunidin; Mv, malvidin. (A),(B),(C) chromatogram represent for corresponding 3 individuals of pEg progeny line. (D),(E),(F) chromatogram represents corresponding 3 individuals of pVm progeny line. (G),(H),(I) chromatogram represents corresponding 3 individual of pCm progeny line. (J),(K),(L) chromatogram represents corresponding 3 individual of pGt progeny line. Each peak was identified by co injection with authentic anthocyanidin standards. X-axis indicate intensity (μV) while U-axis indicate retention time.

Figure S3

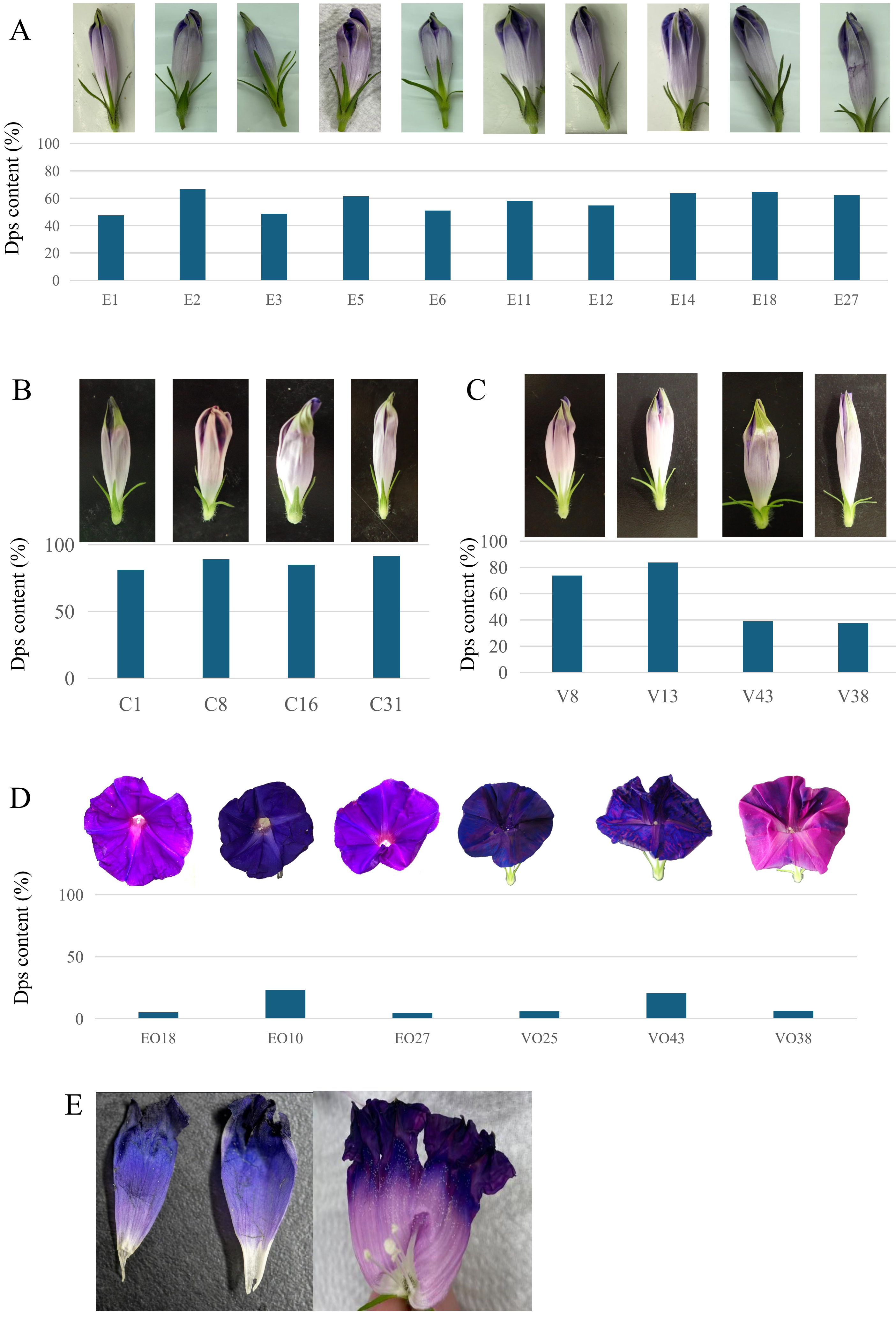

A
Dps content (%)
100
80
60
40
20
0
E1
E2
E3
E5
E6
E11
E12
E14
E18
E27
B
Dps content (%)
100
50
0
C1
C8
C16
C31
C
Dps content (%)
100
80
60
40
20
0
V8
V13
V43
V38
D
Dps content (%)
100
50
0
EO18
EO10
EO27
VO25
VO43
VO38
E

Figure S3. Phenotypic characteristics and pigment accumulation profiles of inhibited and opening *Ipomoea nil* petals. (A–C) Representative images of intact, inhibited T0 of pEg (A), pWv (B) and (C) with the relative Dps content remains highly stable (60% to 85%). (D) Representative images of fully open petals T0 of pEg (EO) and pVw (VO) relative Dps content relatively lower than 25%. (VO43, VO38, EO18, EO27 was sampling from same individual of inhibited petal V43, V38, E18, E27). (E) Structural disintegration of an inhibited pEg petal following attempts to manually force it open. Uneven deformities and malformations at the apical edges of the petals are also characteristic of this severe inhibition.

Fig. S4

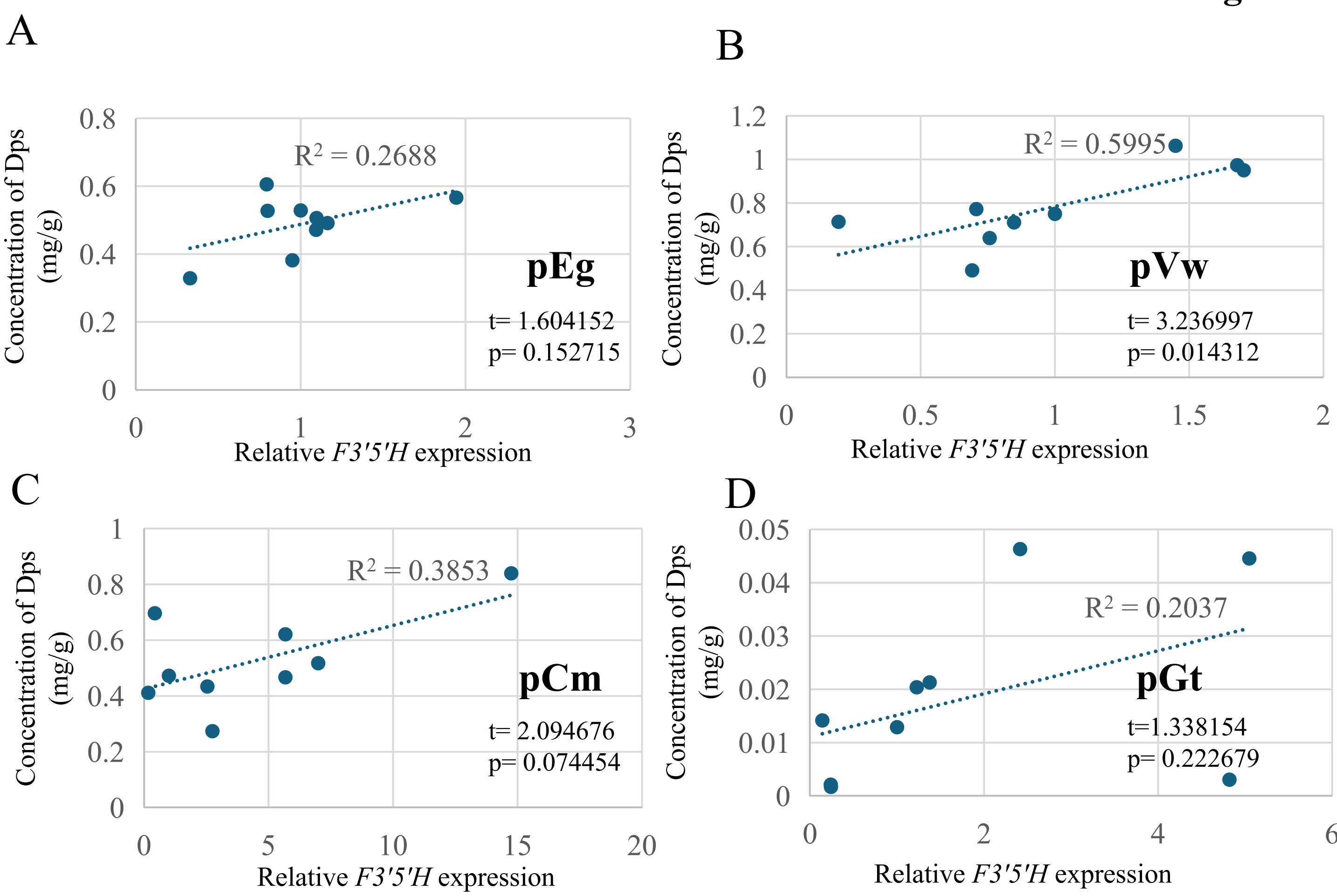

Fig. S4 Correlation analysis between relative *Flavonoid 3′,5′-hydroxylase* (*F3′5′H*) expression and Dps concentration in *Ipomea nil*. Scatter plots showing the linear regression between the relative transcript levels of *F3′5′H* (x-axis) and the concentration of Dps (mg/g) (y-axis). The analysis was performed for four different constructs/lines: (A) pEg, (B) pVw, (C) pCm, and (D) pGt. The dotted blue lines represent the linear trend. The coefficient of determination (R^2), t-value, and p-value are indicated within each panel. (B) indicated that pVw is significant different correlation.

Fig. S5

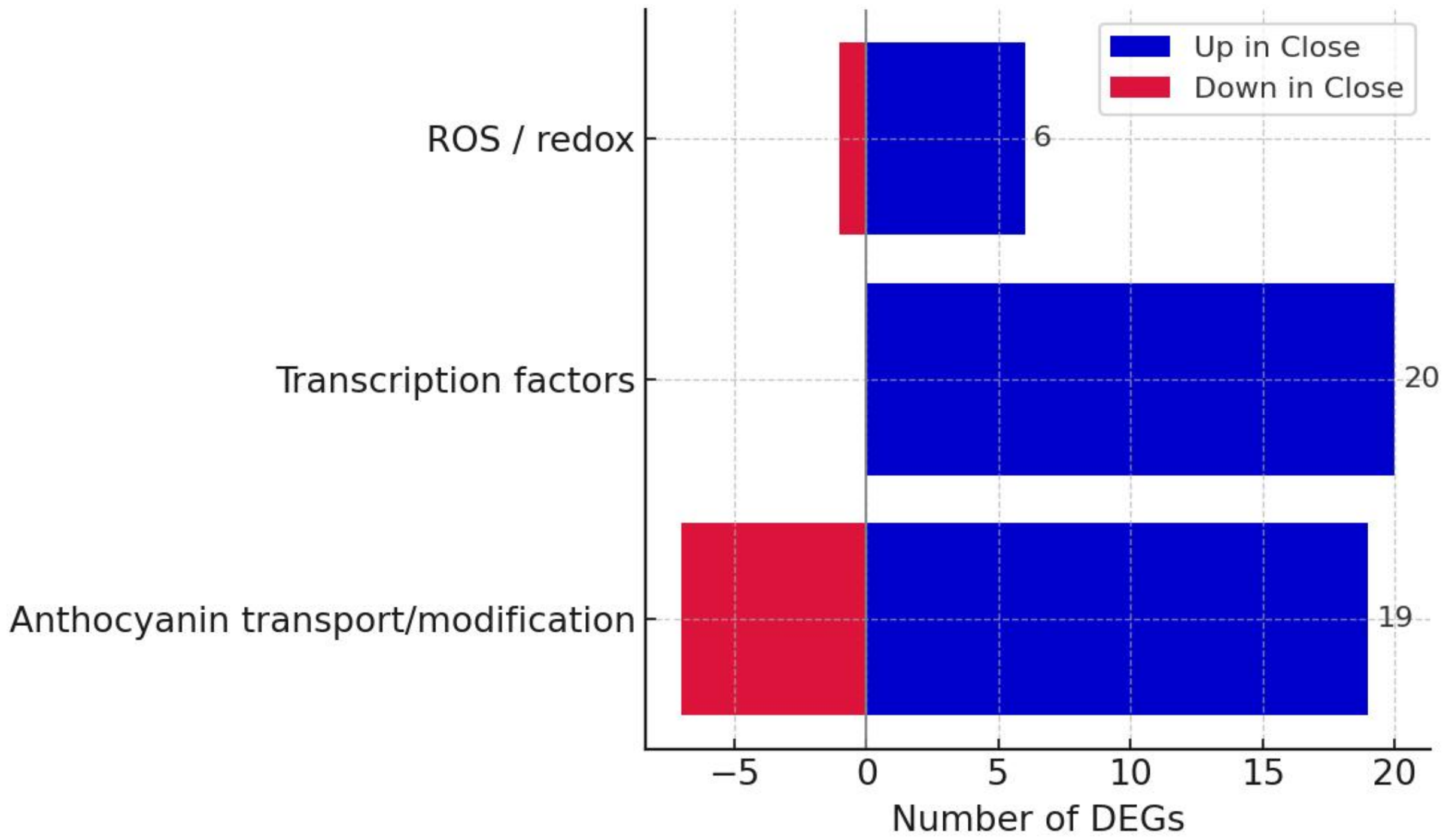

Figure S5. Gene DEGs connect to Anthocyanidin in Inhibited (Close) and Open petals of transgenic *Ipomea nil*. Category-specific distribution of differentially expressed genes (DEGs) between open and closed petals of *I. nil*. The bar plot summarizes the number of DEGs classified into three major functional categories: ROS/redox-related genes, transcription factors, and anthocyanin transport/modification genes. Bars to the right (positive x-axis) represent genes upregulated in closed petals, while bars to the left (negative x-axis) represent downregulated in closed petals. Red for downregulated and blue for up regulated in Inhibited (Close) petal.

Fig. S6

A

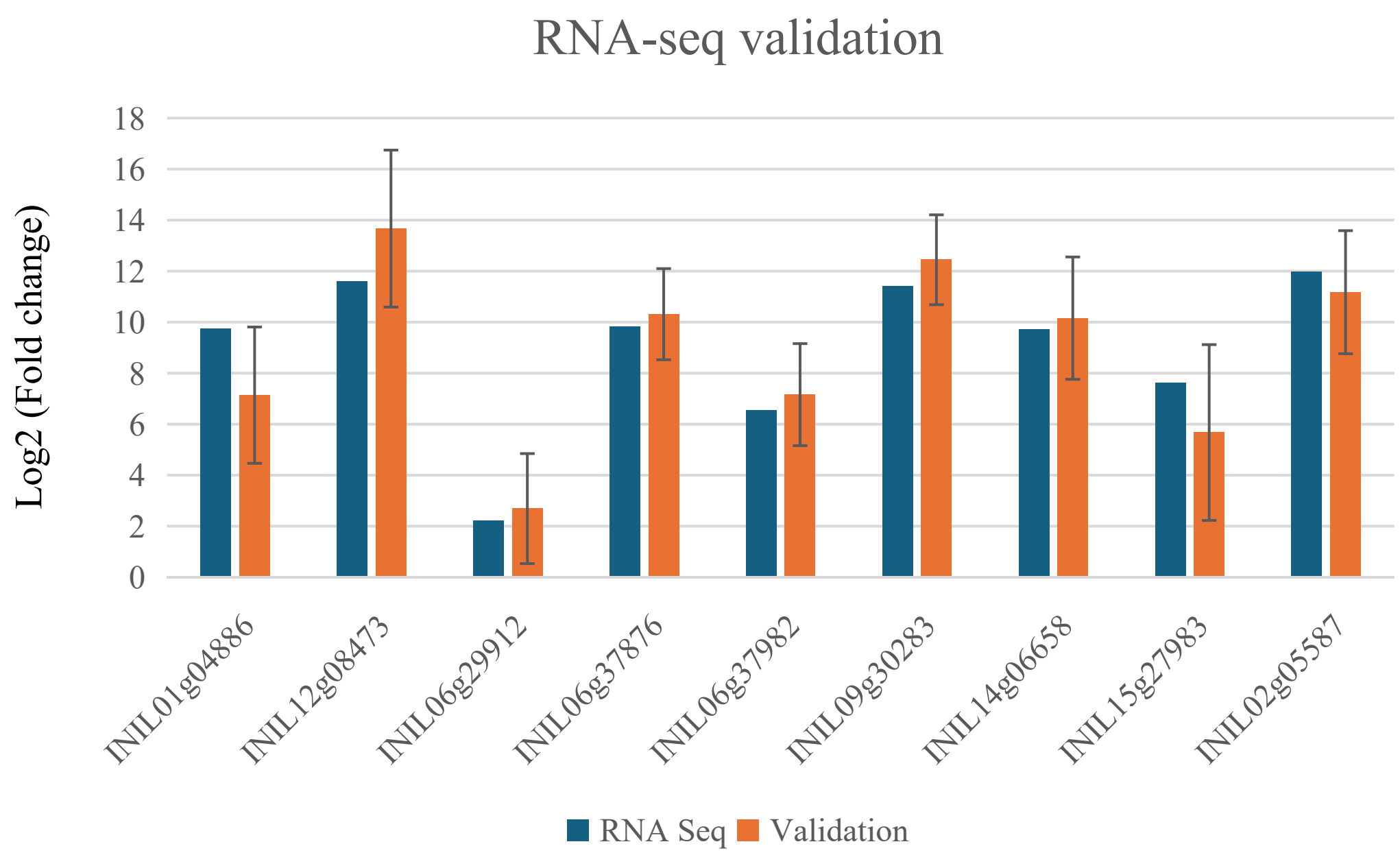

B

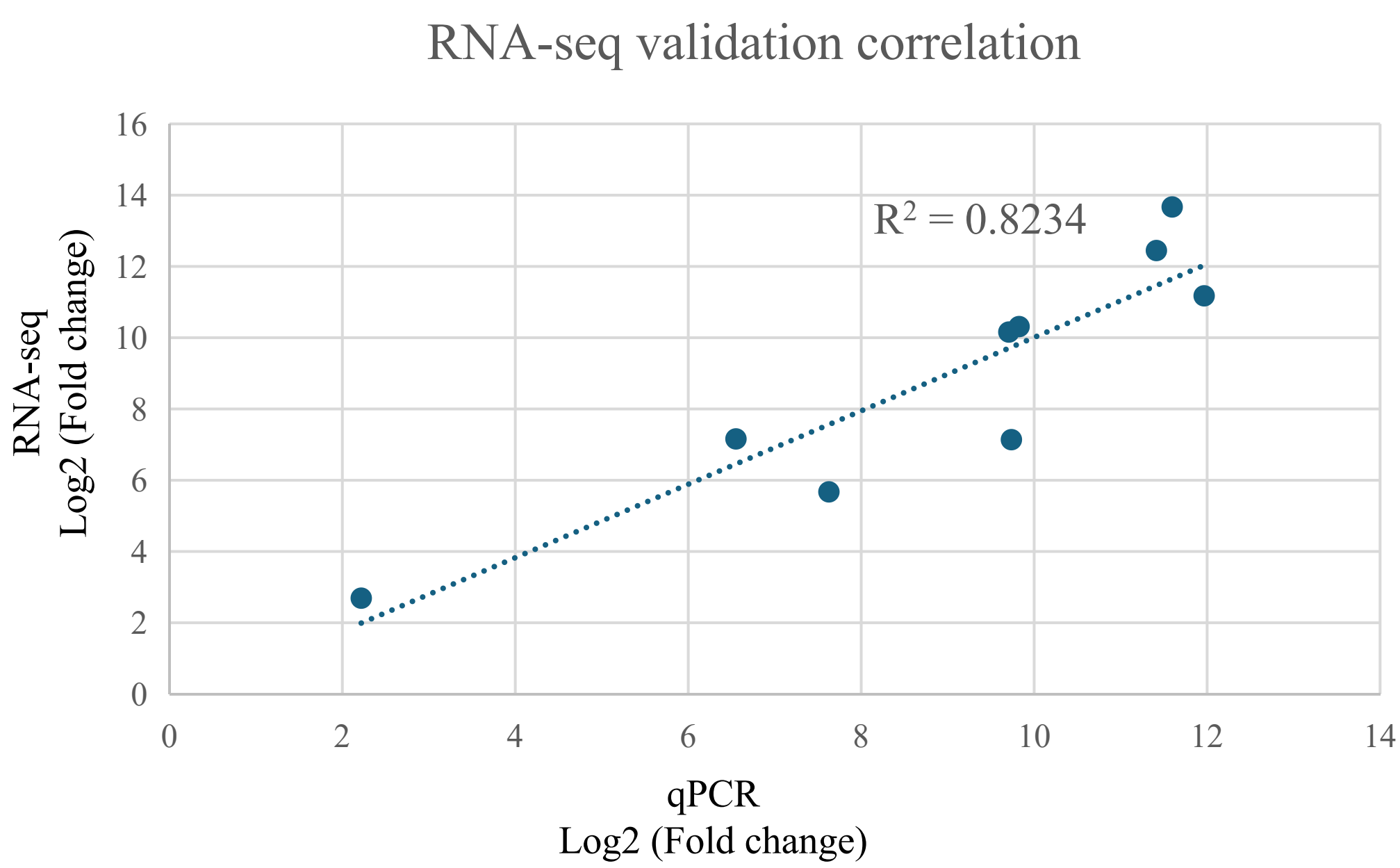

Fig. S6 Validation of DEGs expression between opening and inhibited *Ipomea nil* petals. (A) Comparison of expression levels for selected *I. nil* genes as determined by RNA-Seq (blue bars) and quantitative real-time PCR (orange bars). The x-axis represents the specific gene identifiers, and the y-axis represents the relative expression level and m.value log2 fold change. (B) Plot shows correlation of validation between qRT-PCR and RNA-seq, with linear line representation for regression and R^2 indicates coefficients of determination in correlation between 2 values.

Table S1. Specific primer for amplifying and Infusion of *Eustoma grandiflorum, Campanula medium, Viola wittrockiana,* and *Gentiana trifloral* for constructing transgenic vectors.

**Primers**	**Sequence:5′-3′**	**Comments**
**InFusion-RV-pRI201-F**	CACTGTTGATACATA TGGCTGTTGGAAAT GGCG	Forward primer used for insertion of *EgF3′5′H* in the construction of pEg by In-Fusion cloning.
**InFu RV to pKAN3AH sall R New**	ATTCAGAATTGTCG ACTCAAGGAGAGTA AATATGGAG	Reverse primer used for insertion of *EgF3′5′H* in the construction of pEg or pEg-ADH by In-Fusion cloning.
**InFuCamF35H to pKAN3AH Sal1 F**	CACTGTTGATACATA TGTCTATAGACATA ACCATTCTC	Forward primer used for insertion of *CmF3′5′H* in the construction of pCm
**InFuCamF35H to pKAN3AH Sal1 R**	ATTCAGAATTGTCG ACCTAGACAGTGTA AGCACTTG	Reverse primer used for insertion of *CmF3′5′H* in the construction of pCm
**InFuPansyF35H to pKANre3AH F**	CACTGTTGATACATA TGGCAATTCTAGTCA CCGACTTCGTTG	Forward primer used for insertion of *VwF3′5′H* in the construction of pVw
**InFuPansyF35H to pKANre3AH R**	ATTCAGAATTGTCG ACTCAGGTTGCGTA CGCGTTTG	Reverse primer used for insertion of *VwF3′5′H* in the construction of pVw
**InFuGtF35H to pKAN3AH Sal1 F**	CACTGTTGATACATA TGTCACCCATTTACA CCAC	Forward primer used for insertion of *GtF3′5′H* in the construction of pGt
**InFuGtF35H to pKAN3AH Sal1 R**	ATTCAGAATTGTCG ACTTAAGGAGCATA AACATGAAGAGG	Reverse primer used for insertion of *GtF3′5′H* in the construction of pGt
**InFu RV to Xba-sal cut pKANre**	CACGGGGGACTCTA GATGGCTGTTGGAAA TGGCG	Forward primer used for insertion of *EgF3′5′H* in the construction of pEg-ADH- by In-Fusion cloning..

Table S2. Primer used in qPCR for Transgenic F*lavonoid 3',5'-hydroxylase* (*F3'5'*H) expression level analysis.

	**Forward**	**Reverse**
EgF3'5'H	TTGCCATCCTCTGTGATTGA	GTGGAGTGACCATAGCAGCA
CmF3'5'H	GGATATGTGCAGGGGCTAGA	GCAATGCTATCCCAAAGCTC
VwF3'5'H	GGATGGGAATGGTCCTTGTA	TCAGCGACACCATTTGGTAA
GtF3'5'H	GGGGACATTGGTGCATAGTT	GAATAACCATGGCCGCTAAA
InATP	AACCTCTGCTACTTCATAATTTCTGA	TATGTTGTAGGAGAACCAAGTGCAA

Table S3. Top 20 upregulated gene and comparing to temporal genes expression database of *I. nil.* ↑ (Upregulated), = (Unchange)

Gene Name	Inhibited Petal (vs. control)	Opening Petal vs. Before Opening	Annotated
INIL12g30822	↑	=	GALM
INIL02g05587	↑	=	CjBap12
INIL07g33830	↑	=	Unidentified
INIL12g08473	↑	=	MATE
INIL03g17908	↑	=	HIPP26
INIL09g30283	↑	=	Glycosyl transferase
INIL09g30244	↑	=	60S L18a-like
INIL07g34458	↑	=	Patatin
INIL13g39071	↑	=	Unidentified
INIL06g38254	↑	=	Unidentified
INIL07g14095	↑	=	XTH32
INIL13g08083	↑	=	ASPG2
INIL02g16862	↑	=	Unidentified
INIL07g33831	↑	=	Adenylosuccinate synthase
INIL11g26672	↑	↑	Unidentified
INIL05g22827	↑	=	At4g10955
INIL01g36614	↑	=	WRKY40
INIL06g37875	↑	=	SAG39
INIL12g39991	↑	=	PR2
INIL07g39164	↑	=	RLP12

Table S4. Possible Anthocyanin modification and transporter related related gene of Inhibited (Close) petals compare to Open petals.

GeneID	Note	Family	Regulation	Log2FC	FDR (q-value)	Average Expression	P-Value
INIL14g00674	ABC transporter C family member 2	ABC Transporter	Upregulated	3.653899	0.007845	7.256823	3.18E-06
INIL12g01234	Probable glutathione S-transferase	GST	Upregulated	4.785624	0.014821	4.870148	4.21E-05
INIL04g02722	Zeatin O-glucosyltransferase	Glycosyltransferase	Upregulated	2.112453	0.043586	7.496892	0.000697
INIL11g05198	S-acyltransferase {ECO:0000256|RuleBase:RU079119}	Acyltransferase	Upregulated	2.737045	0.046671	4.2099	0.000862
INIL11g05198	S-acyltransferase {ECO:0000256|RuleBase:RU079119}	Acyltransferase	Upregulated	2.737045	0.046671	4.2099	0.000862
INIL11g05198	S-acyltransferase {ECO:0000256|RuleBase:RU079119}	Acyltransferase	Upregulated	2.737045	0.046671	4.2099	0.000862
INIL13g07713	ABC transporter G family member 25	ABC Transporter	Downregulated	-1.48439	0.047484	8.76045	0.000893
INIL12g08472	MATE efflux family protein {ECO:0000256|RuleBase:RU004914}	MATE Transporter	Upregulated	8.321884	0.009133	3.681916	6.96E-06
INIL12g08473	MATE efflux family protein {ECO:0000256|RuleBase:RU004914}	MATE Transporter	Upregulated	11.59586	0.009133	5.318904	9.90E-06
INIL04g12078	Glutamate--tRNA ligase, cytoplasmic {ECO:0000305}	MATE Transporter	Upregulated	1.659472	0.049338	6.845974	0.00098
INIL10g12890	Probable glutathione S-transferase	GST	Upregulated	4.577479	0.04471	2.811365	0.000756
INIL04g19440	S-acyltransferase {ECO:0000256|RuleBase:RU079119}	Acyltransferase	Upregulated	3.398356	0.02898	5.727264	0.000275
INIL03g21099	ABC transporter A family member 7	ABC Transporter	Upregulated	1.530415	0.033829	7.252844	0.000438
INIL03g21102	ABC transporter A family member 7	ABC Transporter	Upregulated	1.655956	0.035961	8.145255	0.000487
INIL09g21556	Flavonoid 3'-monooxygenase	Other	Upregulated	6.399301	0.046755	5.024224	0.000865
INIL05g24221	7-deoxyloganetic acid glucosyltransferase	Glycosyltransferase	Upregulated	7.59014	0.031817	-2.81853	0.000375
INIL01g25185	Protein S-acyltransferase 8	Acyltransferase	Upregulated	2.161176	0.045002	7.029487	0.000768
INIL01g25272	Glutathione transferase GST 23	GST	Upregulated	3.161046	0.048319	7.392784	0.000925
INIL09g26488	Probable protein S-acyltransferase 12	Acyltransferase	Downregulated	-1.75755	0.02918	8.284249	0.000292
INIL13g26952	Bifunctional 3-dehydroquinate dehydratase/shikimate dehydrogenase, chloroplastic	MATE Transporter	Upregulated	3.105083	0.035086	6.087493	0.000466
INIL12g29111	ABC transporter G family member 15	ABC Transporter	Upregulated	3.07939	0.020354	6.565746	0.000118
INIL04g32780	Leucoanthocyanidin dioxygenase	LDOX	Upregulated	5.858742	0.046343	5.890149	0.000836
INIL07g34529	MATE efflux family protein DTX1	MATE Transporter	Upregulated	3.703061	0.013748	4.55898	2.91E-05
INIL04g34885	Scopoletin glucosyltransferase	Glycosyltransferase	Upregulated	2.760707	0.033915	9.020704	0.00044
INIL14g35270	S-acyltransferase {ECO:0000256|RuleBase:RU079119}	Acyltransferase	Downregulated	-2.33409	0.03318	8.131795	0.000416
INIL09g35842	ABC transporter A family member 2	ABC Transporter	Upregulated	3.166281	0.044111	6.296377	0.000726
INIL09g35940	ABC transporter B family member 25	ABC Transporter	Upregulated	4.637722	0.046324	6.919643	0.000828
INIL09g36108	LRR receptor-like serine/threonine-protein kinase FLS2	FLS	Upregulated	6.255486	0.009133	4.007907	1.11E-05
INIL09g36108	LRR receptor-like serine/threonine-protein kinase FLS2	FLS	Upregulated	6.167461	0.009133	3.651134	1.02E-05
INIL06g37545	ABC transporter C family member 10	ABC Transporter	Downregulated	-2.14	0.040755	7.449071	0.000616
INIL06g38112	UDP-glucose:glycoprotein glucosyltransferase	Glycosyltransferase	Upregulated	2.194689	0.019276	8.329493	0.000107
INIL02g40603	MATE efflux family protein 7	MATE Transporter	Upregulated	3.586771	0.03172	6.945847	0.000372
INIL02g40606	MATE efflux family protein 5	MATE Transporter	Upregulated	8.117673	0.009133	2.57981	9.84E-06
INIL02g40612	MATE efflux family protein 9	MATE Transporter	Downregulated	-1.92814	0.044728	8.671039	0.000757
INIL14g41671	7-deoxyloganetin glucosyltransferase	Glycosyltransferase	Downregulated	-2.84402	0.044111	12.29232	0.000723

Table S5. ROS related Different gene expression of Inhibited (Close) petals compare to Open petals.

Gene ID	m.value	q.value	note
INIL06g37982.t1	6.551426	0.015426	Neutral peroxidase
INIL12g01234.t1	4.785624	0.014821	Probable glutathione S-transferase
INIL06g37985.t1	5.188488	0.033026	Anionic peroxidase
INIL10g12890.t1	4.577479	0.04471	Probable glutathione S-transferase
INIL06g37986.t1	3.68657	0.026904	Anionic peroxidase
INIL05g22724.t1	-2.18991	0.04285	Peroxidase 47

Table S6. Primer list for genes RNA seq validation with qRT-PCR

Gene	Forward Primer	Reverse Primer
INIL06g37982<br>Neutral peroxidase	CACCACCAATTTCACCGGAG	TGGCTAAAACGTCTGCACAC
INIL15g27983<br>PR5	CAAGAACAGGCTGCAATT	CGATGGTGAACTCGGCTAGA
INIL02g05587<br>EG45	ACGAAGATGACCAGTCCAGT	TGGAGGGTGCTGTTTTATGAG
INIL09g30283<br>Glycosyltransferase	TGAGGTGAATGGGAAGAGGC	TATTCTGGGGAGCAGTGTGG
INIL06g37876<br>SAG39	CTGCAACGCCATGAGAAGTG	TCGGTTTCCAGCTTTGTTGA
INIL14g06658<br>WRKY 40	TCGTGGAAGAGGAGGAAGTC	GTCTGGACGTGAACTCTGGA
INIL03g20446<br>CBP60B	GGAAATGGCACGCGTAGATT	GGATACTCACAGTGGCACTT
INIL01g04886<br>SARD1	TGGCAGCAAAACGGATTCAT	GCCAAAGAAGGTCTGATCCG
INIL12g08473<br>MATE	TGTGTCTTTGGTGAATTTCGGT	TCCTTACAACGGACGATTAACA
